# Signalling Alterations in Bones of Pituitary Adenylate Cyclase Activating Polypeptide (PACAP) Gene Deficient Mice

**DOI:** 10.3390/ijms19092538

**Published:** 2018-08-27

**Authors:** Gergő Józsa, Vince Szegeczki, Andrea Pálfi, Tamás Kiss, Zsuzsanna Helyes, Balázs Fülöp, Csaba Cserháti, Lajos Daróczi, Andrea Tamás, Róza Zákány, Dóra Reglődi, Tamás Juhász

**Affiliations:** 1 Department of Anatomy, MTA-PTE PACAP Research Team, University of Pécs Medical School, Szigeti út 12, H-7624 Pécs, Hungary; dr.jozsa.gergo@gmail.com (G.J.); fulopbalazs87@gmail.com (B.F.); andreatamassz@gmail.com (A.T.); dora.reglodi@aok.pte.hu (D.R.); 2 Department of Anatomy, Histology and Embryology, Faculty of Medicine, University of Debrecen, Nagyerdeikrt. 98, H-4032 Debrecen, Hungary; szeg.vince@gmail.com (V.S.); p_andi@hotmail.hu (A.P.); roza@anat.med.unideb.hu (R.Z.); 3 Department of Pharmacology and Pharmacotherapy, University of Pécs Medical School, Szigeti út 12, H-7624 Pécs, Hungary; kiss891012@gmail.com (T.K.); zsuzsanna.helyes@aok.pte.hu (Z.H.); 4 Department of Solid State Physics, University of Debrecen, Bem tér 18/b, H-4026 Debrecen, Hungary; cserhati.csaba@science.unideb.hu (C.C.); lajos.daroczi@science.unideb.hu (L.D.)

**Keywords:** hedgehog, BMP, collagen expression, inorganic matrix, alkaline phosphatase activity, bone fragility

## Abstract

Pituitary adenylate cyclase activating polypeptide (PACAP) is a neuropeptide with diverse developmental roles, including differentiation of skeletal elements. It is a positive regulatory factor of chondrogenesis and osteogenic differentiation in vitro, but little is known about its *in vivo* role in bone formation. In our experiments, diaphyses of long bones from hind limbs of PACAP gene-deficient mice showed changes in thickness and increased staining intensity. Our main goal was to perform a detailed morphological and molecular biological analysis of femurs from PACAP knockout (KO) and wild type (WT) mice. Transverse diameter and anterior cortical bone thickness of KO femurs showed significant alterations with disturbed Ca^2+^ accumulation and collagen type I expression. Higher expression and activity of alkaline phosphatase were also observed, accompanied by increased fragility PACAP KO femurs. Increased expression of the elements of bone morphogenic protein (BMP) and hedgehog signalling was also observed, and are possibly responsible for the compensation mechanism accounting for the slight morphological changes. In summary, our results show that lack of PACAP influences molecular and biomechanical properties of bone matrix, activating various signalling cascade changes in a compensatory fashion. The increased fragility of PACAP KO femur further supports the role of endogenous PACAP in *in vivo* bone formation.

## 1. Introduction

Molecular mechanisms regulating bone development and regeneration have been intensely studied over the last two decades, but still there are several signalling pathways with less-understood functions in these fields [1]. Morphology and stability of long bones determine the size, strength and motility of animals. Subsequently, the maintenance of a proper inorganic and organic matrix composition and turnover is essential for the appropriate biomechanical function of long bones [2]. Matrix production and its composition are crucial factors for adequate limb development and normal callus formation [3]. Development of long bones or the osteoprogenitor-osteoblastic transformation is regulated by various signalling mechanisms such as bone morphogenic protein (BMP), hedgehog (HH) and Wingless int1 (WNT) pathways [1,4]. The initial steps of osteogenesis are followed by the induction of bone specific matrix production, in which collagen type I is the major organic component. Orientation and thickness of collagen lamellas together with inorganic components provide the specific architecture in cortical bone. This lamellar architecture ensures the biomechanical stability and the tensile strength of compact bone [5]. Besides the production of the organic components of bone matrix, osteoblasts also take an active part in the calcification of bone matrix. Calcium hydroxyapatite deposits between the collagen lamellas and intercalation of calcium salt crystals in the periodic gaps of collagen fibrils provide the hardness of bone tissue [6].

For precise regulation of extracellular matrix (ECM) production, activation of osteoblasts requires a proper timing and milieu which can be triggered by cytokines, growth hormones or other endocrine/paracrine pathways [1]. BMP is one of the basic activators of bone development which can bind to BMPR (bone morphogenic protein receptor) and trigger the phosphorylation of Smad1/5 followed by its nuclear translocation. This pathway induces the production of various proteins such as alkaline phosphatase (ALP), osterix or even collagen type I; all are essential to proper bone formation [7]. Furthermore, it has been demonstrated that BMPs activate the transcription factor cyclic adenosine monophosphate (cAMP) response element-binding protein (CREB) via the activation of protein kinase A (PKA) which also triggers the expression of molecules mentioned above [8]. On the other hand, bone formation is regulated by Runx2 transcription factor which can also be activated by PKA and it regulates the expression of bone specific molecules and ECM components [9]. At this point there is a putative link between pituitary adenylate cyclase activating polypeptide (PACAP) signalling and bone formation, as PKA is its first described and major intracellular mediator [10].

PACAP comprises 38 amino acid residues (PACAP 1-38) and a shorter α-amidated form that corresponds to the 27 residues of the N-terminus (PACAP 1-27) can also be generated. Its biologically active region is completely preserved during evolution [11]. The presence of the neuropeptide has been shown in several peripheral tissues, such as in gonads [12], intestine [13], kidney [14], or testis [15]. It has been demonstrated that PACAP can prevent the harmful effect of oxidative stress [16,17], trigger the activation of anti-inflammatory processes and can have a positive role during ischemic conditions [18]. PACAP is also known to activate the immune system [19] and to induce tissue regeneration [20]. It plays an important role in the development of different organs, regulates chondrogenesis and spermatogenesis [16], and influences the development of the central nervous system [10,21]. Numerous recent experiments have also proven the importance of PACAP in limb development [16,22,23].

PACAP has three major receptors, vasoactive intestinal polypeptide receptor (VPAC)1, VPAC2 and pituitary adenylate cyclase-activating polypeptide type I receptor (PAC1), the latter one of which has the highest affinity to the neuropeptide [10]. PAC1 and VPAC1 receptors have been proven to be expressed on UMR-106 osteoblast cells [24], while VPAC2 was detected in MC3T3E1 calvarial osteogenic cell lines [25]. Release of PACAP from sympathetic nerve endings in bone tissue has also been demonstrated [26]. In vitro it has a positive effect on calcification and bone matrix production [16,24]. PAC1 receptor is a G protein coupled receptor, which has several crosstalks with different signalling pathways [10,22]. Binding of PACAP receptors leads to the induction of PKA activation, the canonical signalling pathway of PACAP signalization. PKA can subsequently activate several transcription factors such as CREB, Runx2 or Sox9 [16,22,24]. The activation of PACAP receptors may form a signalization crosstalk with BMP, WNT or β-catenin pathways or have a negative regulatory connection with the Sonic Hedgehog (SHH) signalization [27]. Ca^2+^ dependent pathways can be activated via PAC1 receptors and intracellular Ca^2+^ release has been detected in the presence of PACAP [28].

The importance of PACAP and its signalization has been proven in several tissues, but we do not have a clear picture about its role in long bone development. In this study we demonstrate the importance of PACAP in the regulation of matrix production in long bones. Furthermore, we analyzed the possible signalling crosstalk in wild type (WT) and PACAP KO mice, possibly modifying the biomechanical stability of cortical bone.

## 2. Results

### 2.1. Bone Morphology Is Only Slightly Modified in PACAP Gene Deficient Mice

**Figure 1:**
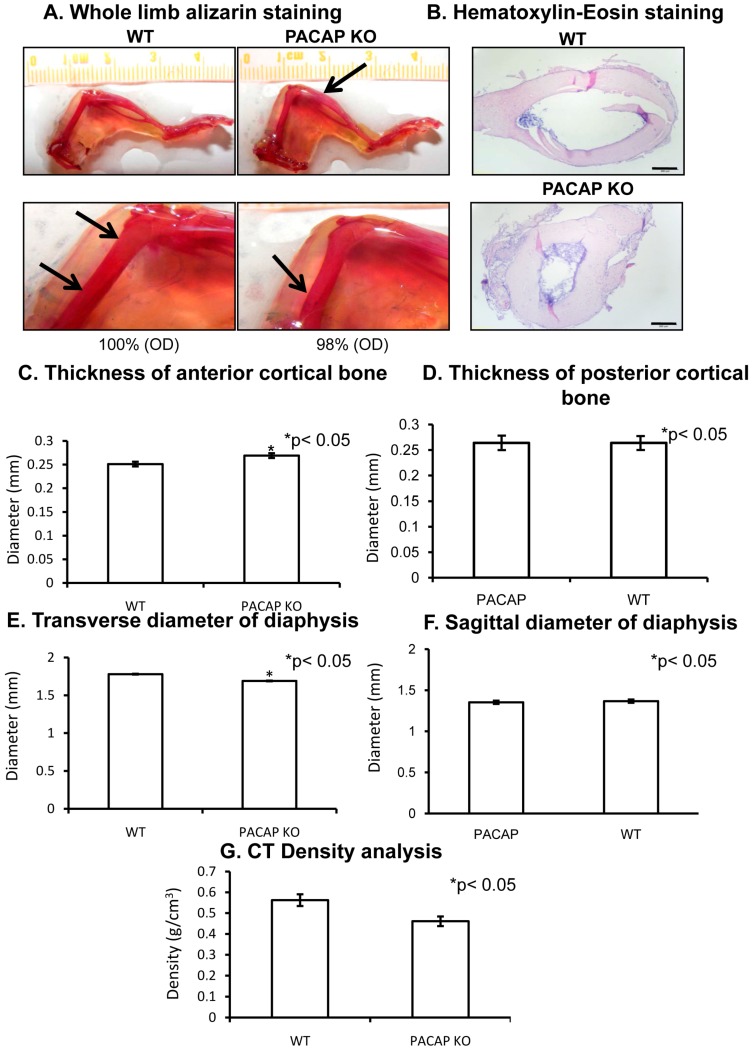
Morphological analysis of hind limbs of wild type (WT) and pituitary adenylate cyclase activating polypeptide (PACAP) knockout (KO) mice. Whole limb alizarin red staining (**A**), optical density (OD) was determined in 1 cm distal part of the femur. Hematoxilin-eosin (HE) staining (**B**) to visualize the histological differences. Original magnification was 4×. Scale bar, 500 µm. CT analysis (**C**–**G**) of mouse femurs. Representative data of 5 independent experiments. Asterisks indicate significant (* *p* < 0.05) difference in thickness of cortical bone or in the diameter of diaphysis compared to the respective control.

First, anatomical alterations were investigated in hind limbs of PACAP gene-deficient mice. Interestingly hind limb bone morphology of PACAP KO mice was not significantly altered. No differences were detected in the length of femur or tibia, and no other anatomical alterations were visible (Figure 1A and Figure S3). No difference was found in the length of tibia with CT (computed tomography) analysis. Femur length of PACAP KO mice was tendentiously shorter but did not reach a significant level (Figure S2A,B). Randomly increased alizarin red intensity was detected as it is visible at the tip of arrows in Figure 1A. However, some thinner parts also appeared in the femur diaphyses of gene deficient mice (Figure 1A), but the optical density of the stained bone had no significant alterations in PACAP KO animals (Figure 1A and Figure S3). Therefore, further analysis was performed focusing on femurs. In cross sections of the diaphyses, the cortical part was thicker in PACAP KO animals than in WTs with hematoxilin-eosin (HE) staining (Figure 1B). The diameter of bone marrow cavities was wider in WT animals (Figure 1B) in sections from the same level. A CT analysis was performed to quantify the exact differences of cortical bone thickness and internal cavity. The proximal and distal epiphyses did not show any significant morphological differences, therefore only the diaphyses were further analyzed. Femur lengths were not significantly altered in PACAP KO mice (Figure S2A). Similarly, no significant differences were found in the posterior cortical area or in the sagittal diameters (Figure 1D). On the contrary, the anterior cortical bone was significantly thicker in the gene deficient animals (Figure 1C). Although the sagittal diameter of the diaphyses had no significant alterations, the transverse diameters were significantly shorter in PACAP KO mice (Figure 1E,F). Further analysis of the CT results did not show any other significant alterations, only the density of the femur at the level of greater trochanter was tendentiously lower in PACAP KO animals (Figure 1G), suggesting certain alterations in the bone matrix of KO animals.

### 2.2. Inorganic Matrix Production Is Disturbed in PACAP KO Mice

**Figure 2:**
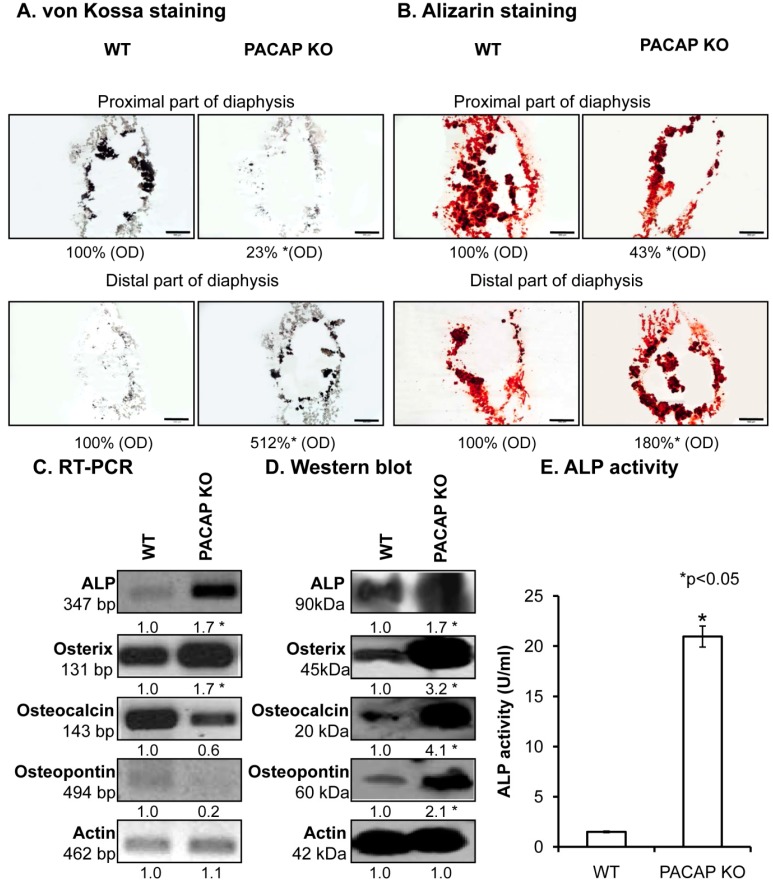
Investigation of inorganic matrix production of long bones. von Kossa (**A**) and alizarin red (**B**) staining of cryosectioned samples. Original magnification was 4×. Scale bar: 500 µm. Optical density (OD) was determined in microphotographs and normalised to wild type samples. mRNA (**C**) and protein (**D**) expression of alkaline phosphatase (ALP), osterix, osteocalcin and osteopontin of femurs. For reverse transcription followed by polymerase chain reaction (RT-PCR) and Western blot reactions, actin was used as control. Optical signal density was measured and results were normalised to the controls. For panels (**C**,**D**) numbers below signals represent integrated signal densities determined by ImageJ software. (**E**) ALP activity in compact bone. Asterisks indicate significant (* *p* < 0.05) alteration of ALP activity compared to the respective control. Representative data of 3 independent experiments.

In further steps, the inorganic matrix component of femur diaphyses was investigated in detail. To visualize the Ca^2+^ content of bone ECM von Kossa and alizarin red stainings were performed. A remarkable inhomogeneity in Ca^2+^-phosphate content of the diaphyses in certain parts of the KO femurs was observed (Figure 2A). The femurs contained more Ca^2+^ phosphate closer to the distal end of the diaphyses, while the proximal parts had reduced Ca^2+^ content in KO compared with WT mice (Figure 2A and Figure S4A). Although these differences were visible with alizarin red staining, the whole femur did not have any macroscopical disorders. In order to explore the possible background molecular mechanisms, expression levels of enzymes responsible for matrix production in bone were followed with molecular biological methods. mRNA expression was altered (Figure 2C and Figure S4B) and protein expressions of alkaline phosphatase (ALP) and its target molecule, osterix, were significantly elevated in PACAP deficient animals (Figure 2D and Figure S4C). Moreover, other matrix molecules, such as osteocalcin and osteopontin, which can be related to the altered inorganic bone matrix deposition, showed increased expression in KO mice (Figure 2D and Figure S4C). Their mRNA expression did not show alterations (Figure 2C and Figure S4B). As protein expression is not always in direct relation with the enzyme activity, ALP activity was also assayed and a dramatic increase was detected in femur samples of PACAP KO mice (Figure 2E).

### 2.3. Augmented Collagen Type I Expression in PACAP KO Mice

**Figure 3:**
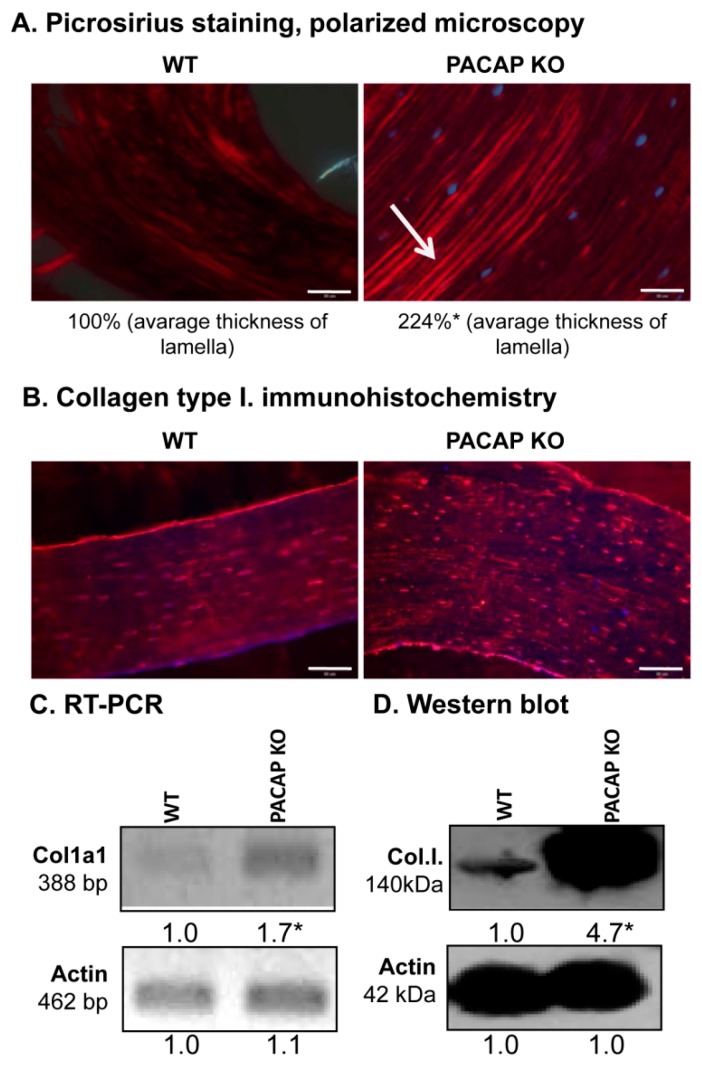
Collagen type I expression in femurs. (**A**) Collagen expression of cortical bone was visualized with picrosirius staining, and photomicrographs were made by polarization microscope. Original magnification was 20×. Scale bar: 200 µm. Numbers below the photos show the differences of concentric lamellas thickness. (**B**) Immunohistochemistry of collagen type I in cortical bones. Original magnification was 20×. Scale bar: 200 µm. mRNA (**C**) and protein (**D**) expression of collagen type I. For RT-PCR and Western blot reactions, actin was used as controls. Optical signal density was measured and results were normalised to the controls. For panels (**C**,**D**) numbers below signals represent integrated signal densities determined by ImageJ software. Asterisks indicate significant (* *p* <0.05) alteration of expression compared to the respective controls. Representative data of 3 independent experiments.

Morphology of the femur was not altered in spite of the elevated inorganic bone matrix production, therefore, further analysis of organic matrix production was performed. Orientation of collagen type I-made fibers in a cortical lamella is a crucial factor in determining the microscopical structure and macroscopic morphology of compact bone. Therefore, this organic component of compact bone was further investigated. Concentric lamellae of compact bone were found thicker in PACAP gene deficient mice shown with picrosirius red staining in polarization microscope (Figure 3A). Collagen fibers in WT animals were present, although they seemed to be thinner (Figure 3A and Figure S5A). Furthermore, the immunohistochemical analysis verified the previous results (Figure 3B and Figure S5B): concentric collagen lamellas were visualised in both experimental groups with stronger signals of PACAP KO mice (Figure 3B). Immunopositivity around the osteocytes had no significant differences between WT and KO mice (Figure 3B and Figure S5B). To detect the total expression of collagen type I, reverse transcription followed by polymerase chain reaction (RT-PCR) and Western blot were performed. mRNA expression of the major organic extracellular matrix component was elevated in PACAP gene deficient animals (Figure 3C and Figure S5C), and a dramatically increased protein expression was also observed (Figure 3D and Figure S5D).

### 2.4. Alterations of PACAP Signalling Pathway in Cortical Bone

**Figure 4:**
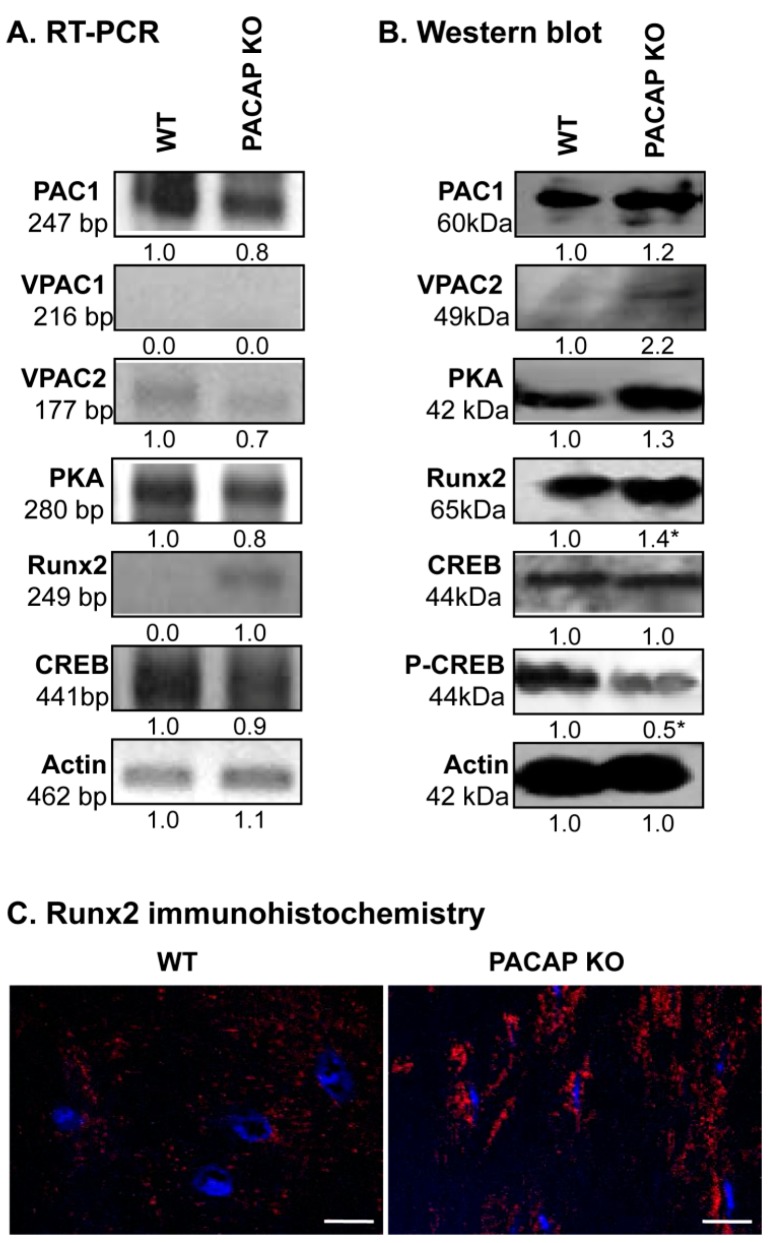
PACAP signalisation in bones. (**A**) mRNA and (**B**) protein expression of pituitary adenylate cyclase-activating polypeptide type I receptor (PAC1), vasoactive intestinal polypeptide receptor (VPAC)1, VPAC2,protein kinase A (PKA), cyclic adenosine monophosphate (cAMP) response element-binding protein (CREB), Runx2 in cortical bone. Actin was used as a control. Numbers below signals represent integrated signal densities determined by ImageJ software. Asterisks indicate significant (* *p* < 0.05) alteration of expression compared to the respective controls. Representative data of 3 independent experiments. (**C**) Immunohistochemistry of Runx2 in cortical bones. Original magnification was 60×. Scale bar: 5 µm. Representative data of 3 independent experiments.

As PACAP can act on three different receptors, we monitored mRNA and protein expressions of PAC1, VPAC1 and VPAC2 receptors. We were not able to detect the mRNA of VPAC1 receptor in cortical bone (Figure 4A). PAC1 receptor was expressed equally in PACAP KO and WT mice, while the protein expression of VPAC2 was elevated in gene deficient animals (Figure 4A,B and Figure S6A,B). The major downstream target of PACAP signalization is PKA, which was detected in both genotypes without significant differences in its expression (Figure 4A,B and Figure S6A,B). The most important transcription factors that are substrates of PKA, are CREB and Runx2 in bone development. Phosphorylation of CREB is regulated by PKA and a reduced amount of phospho-CREB was detected in PACAP KO mice, while mRNA expression of CREB was detected without alteration and the unphosphorylated protein of CREB was not altered significantly (Figure 4A,B and Figure S6A,B). Surprisingly, the mRNA and protein expression of Runx2 transcription factor was increased in bone samples of PACAP deficient mice (Figure 4A,B and Figure S6A,B). Moreover, a much higher level of nuclear Runx2 was detected in osteocytes of KO femurs (Figure 4C and Figure S6C).

### 2.5. Alterations of Possible Signalling Crosstalks

**Figure 5:**
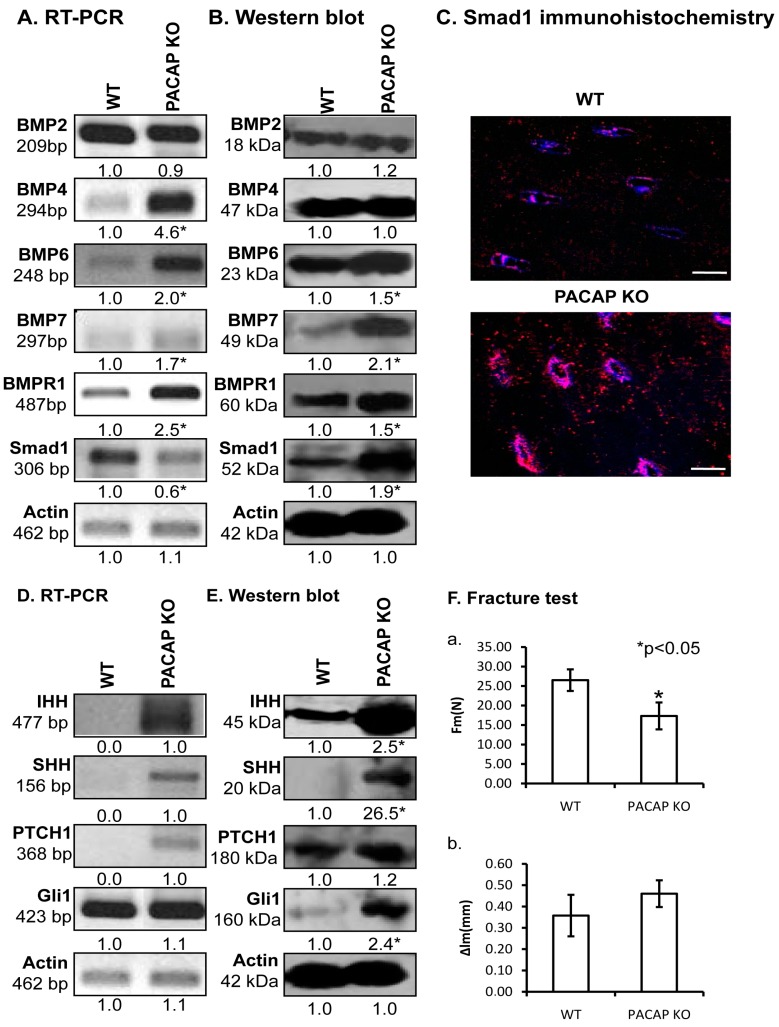
Signalling connections of PAC1 receptor. mRNA (**A**,**D**) and protein (**B**,**E**) expression of bone morphogenic protein (BMP)2, BMP4, BMP6, BMP7, BMPR1, Smad1, Indian Hedgehog (IHH), Sonic Hedgehog (SHH), Patched (PTCH)1 and Gli1 in cortical bone. For RT-PCR and Western blot reactions, actin was used as controls. Signals optical density was measured and results were normalised to the optical density of controls. For panels (**A**,**D**) and (**B**,**E**) numbers below signals represent integrated densities of signals determined by ImageJ software. (**C**) Smad1 immunohistochemistry in bones. Original magnification was 60×. Scale bar: 5 µm. (**F**) Three point bending tests of long bones. (**a**) Value of the loading force at crack (F_m_), and (**b**) the value of the bending deformation at crack (Δl_m_) were demonstrated. Asterisks indicate significant (* *p* < 0.05) alteration of expression as compared to the respective control. Representative results of 3 independent experiments.

One of the major regulators of bone development is the BMP signalling cascade. Therefore, we investigated the ligands of BMPR1, which is known to play a crucial role in limb development [4]. No alterations were detected in the mRNA or protein expressions of BMP2 (Figure 5A,B and Figure S7A,B). Similarly, BMP4 protein expression had no difference between the two different genotypes, although BMP4 mRNA expression was tendentiously elevated in KO animals (Figure 5A and Figure S7A,B). BMPs, such as BMP6 and BMP7, playing fundamental roles in cortical bone formation, showed increased mRNA and significant protein expression elevation in PACAP KO mice (Figure 5A,B and Figure S7A,B). Furthermore, BMPR1, the receptor of these BMPs showed higher mRNA and protein expression in the PACAP KO animals than in WT mice (Figure 5A,B and Figure S7A,B). Activation of BMP signalling can be judged by its downstream targets, such as Smad transcription factors. Although mRNA expression of Smad1 was tendentiously reduced in gene deficient mice (Figure 5A and Figure S7A), the protein expression was significantly elevated, almost doubled in the same samples (Figure 5B and Figure S7B). Smad1 nuclear localization is a sign of its activation, therefore, immunohistochemistry was performed. In the cortical bone of PACAP KO mice the nuclear localization of Smad1 was stronger in confocal series than in WT littermates (Figure 5C and Figure S7C).

PACAP activation exerts an inhibitory effect on hedgehog signalling, which in turn influences extracellular matrix production and BMP expression. We monitored the molecular components contributing in the canonical way of hedgehog signalization. Both the mRNA and protein expression of Indian Hedgehog (IHH) and SHH increased in PACAP gene deficient bones (Figure 5D,E and Figure S7A,B). Likewise, the main receptor of these ligands, Patched (PTCH)1 also increased in the PACAP KO animals (Figure 5D,E and Figure S7A,B), and mRNA and protein expression of Gli1, the downstream target of the receptor activation, was elevated in gene-modified tissues (Figure 5D,E and Figure S7A,B).

### 2.6. Biomechanical Properties of Bones Are Altered in PACAP KO Mice

As we observed significantly altered BMP and HH signalization and imbalanced bone matrix composition in bone samples of PACAP KO mice without any obvious macroscopic morphological changes, we hypothesized that the bone tissue in KO femurs may have worse biomechanical properties compared to WT samples. Therefore, we performed bone fracture assays and measured two parameters. The force needed for a complete fracture at shafts of PACAP KO femur was significantly lower than in WT mice (Figure 5(Fa)). Reflection of the femur shafts had no significant differences between the two genotypes, although the bones from PACAP deficient mice had tendentiously higher inclination (Figure 5(Fb)).

## 3. Discussion

PACAP is a neuropeptide with important roles in several differentiation processes in the central nervous system and also in the periphery [10,29,30]. Our laboratory has already proven its importance during in vitro cartilage differentiation and we have also demonstrated its preventative function during oxidative stress [16] and mechanical load in chondrogenesis [27]. Presence of PACAP positive sympathetic fibers was detected in the epiphysial cartilage canals of pigs and as a neurohormone it can also reach bones via vessels [26]. In vitro studies have proven the presence of PACAP receptors in MC3T3E1 [25] and UMR 106 osteoblastic cell lines [24]. Although PACAP gene deficient mice do not have marked visible morphological alterations, their behavior and movement activity is slightly changed compared with the wild type animals [31].

In our experiments, we found only minor macroscopic differences in bone shape and size but the detailed CT analyses showed an increase of anterior cortical bone thickness and elevation of transverse diameter in the femur shafts in KO mice. The density of femur trochanter did not show significant alterations but it was tendentiously lower in PACAP gene deficient mice. As significant changes were detected mostly in the femur, therefore our main experiments were focused on this bone. Disorders of femur signalling pathways result in an alteration of bone microarchitecture, subsequently, fracture characteristics and mechanical load bearing of long bones may decrease [32,33].

**Figure 6:**
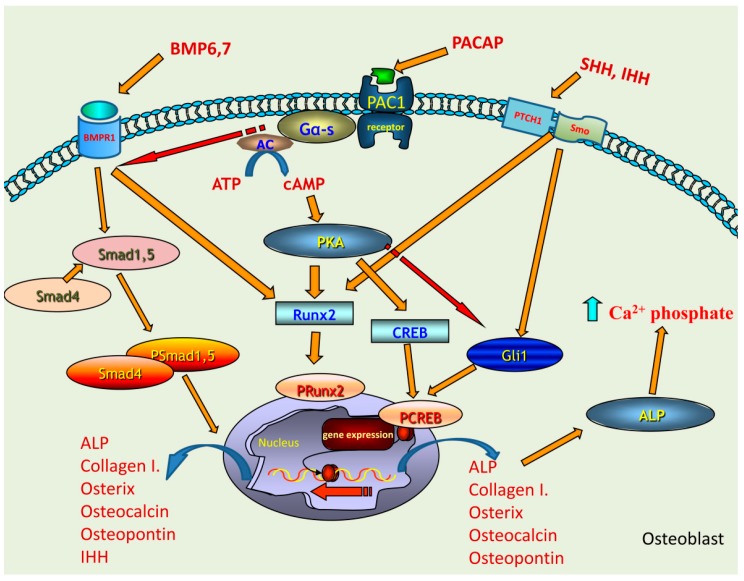
Schematic drawing of the possible signalling pathways regulated by PACAP in bone formation. PAC1 receptor activation leads to the increase of intracellular cAMP, which activates PKA. Downstream targets of PKA can be CREB or Runx2 transcription factors which activations induce the expression of collagen type I, osterix, osteocalcin, ostepontin and ALP. BMP6 and BMP7 binding to BMPR1 triggers the activation of Smad cascade which can induce the expression of collagen type I, osterix, osteocalcin, ostepontin, ALP and IHH. The activation of BMP signalling is fine-tuned by the activation of PACAP signalling. Runx2 phosphorylation can occur via BMPR1 activation, showing a PACAP independent mechanism. IHH and SHH activate Gli1 transcription factor which is inhibited by PACAP signalling. This signalling pathway is also responsible for the activation of collagen type I and ALP expression. Increased ALP expression and activation elevates the Ca^2+^ phosphate production. PACAP signalling keeps a balance between BMP and hedgehog signalling activation via this regulates proper bone formation.

To investigate the molecular mechanisms in the background of cortical bone histological alterations, we focused on the signalling pathway of PACAP and putative signaling crosstalks. It has been demonstrated that the lack of Gα protein reduces bone mass in mice [34]. As PAC1 receptor is a G protein coupled receptor, its inactivation may be involved in the generation of an altered bone architecture. Since significant morphological changes were not visible at KO femurs, we hypothesized that the lack of PACAP signalling induced activation of unusual crosstalk of certain signalling molecules or evoked compensatory molecular mechanisms. PAC1 receptor was detected in WT and PACAP KO mice equally but the expression of its downstream elements was altered. Activation of PACAP receptors induces the elevation of intracellular cAMP concentration, subsequently triggering elevated activation of PKA, which is considered the classical downstream mechanism of PACAP signaling [10]. PKA can phosphorylate several transcription factors in skeletal tissue development, such as CREB, Sox9 [35,36] or Runx2 [24]. The nuclear translocation of these factors induces the activation of genes encoding extracellular matrix component proteins. The expression of PKA and CREB did not significantly change, but the more active, phosphorylated form of CREB decreased in gene deficient mice. Activation/phosphorylation of CREB is required for proper bone formation [37], but there are other transcription factors which can also be influenced by PKA signalling pathway and contribute to the regulation of osteogenesis. Runx2 can be activated via PACAP-PKA axis [24], which was increased in PACAP KO mice. This may suggest a PACAP dependent and independent activation of this transcription factor which may compensate the lack of CREB activation. On the other hand, Runx2 via the IHH activation also plays a role in ossification [38]. Both CREB and Runx2 can regulate the expression of collagenous and non-collagenous proteins building organic bone matrix and influencing deposition of inorganic components of compact bone (Figure 6).

First the inorganic component of the extracellular matrix of femur shafts was investigated in detail. We found focal and regional heterogeneity in the staining intensity of Ca^2+^ positive matrix areas in femur diaphyses of PACAP KO mice. These findings suggested disturbed ossification and local architectural disorders of cortical bone, which may result in altered biomechanical properties of the femurs of PACAP gene deficient animals [39]. In line with these findings, we reported structural changes in hard tissues of the teeth of PACAP KO mice [40]. Another finding supporting disturbed bone mineralisation was the significantly elevated protein expression and activity of ALP in cortical bone tissue samples of PACAP KO femurs. Expression of bone matrix non-collagenous proteins such as osterix and osteocalcin, which play a substantial role in inorganic matrix binding to the organic matrix, was also found elevated in the same samples. Bone specific ALP is one of the most important signalling elements which can determine the matrix composition of cortical bone [41]. Although we did not investigate the tissue distribution of these proteins, but heterogeneity in the staining intensity of the calcified matrix suggests that the above bone matrix proteins are probably not equally distributed in the cortical bone of femurs, resulting in focal matrix deposition disorders. Similar phenomena appear during aging or mechanical loading in bone matrix production [42]. Induction of the transcription of *ALP* gene can occur via Runx2 [43], and we observed that expression and nuclear localization of this transcription factor was significantly higher in gene deficient mice.

Subsequently, ALP activation can affect collagen secretion by osteocytes and osteoblasts [41]. In gene-deficient mice we detected an elevated collagen type I expression and altered lamellar structure in compact bone. The increased accumulation of collagen type I can be a good marker of biomechanical characteristics of long bones [44]. Collagen type I has special spiral orientation in cortical bone lamellae. Alterations in collagen fiber orientation in lamellae may have an effect on strength of bone or on callus formation which strongly influences bone stability and biomechanical properties [6]. In chronic arthritis the expression and remodelling of collagen type I is altered, which results in an increased fragility of bones [45]. Taken together, the proper orientation and expression level of collagen type I is essential for the stability of long bones. Indeed, when we investigated whether these alterations in bone matrix production had any negative effect on biomechanical properties of shafts of KO femurs we found that significantly lower force was needed for bone fracture examining KO bones.

As both the organic and inorganic components of bones were altered without major morphological disorders, some compensatory changes in the activity of various regulatory mechanisms are supposed, which are physiologically influenced by PACAP signalling. We have shown a PACAP induced Runx2 nuclear accumulation in UMR-106 cell line [24]. PACAP deficient mice also showed an elevated nuclear presence of Runx2, which suggests a PACAP independent activation (Figure 6). It is known that osteogenic differentiation can be regulated by BMP/Runx2/osterix axis as BMP2 can activate Runx2, which in turn can regulate osterix expression [9]. PACAP signalisation exerts effects on several, putatively crosstalking downstream signalling pathways, such as WNT, β-catenin, hedgehog and BMP [22,27]. First, we focused on BMP signalling and found elevated mRNA expression of BMP4, 6 and 7, while protein levels only of BMP6 and 7 were higher in PACAP KO mice. No alterations were shown in either the mRNA or protein expression of BMP2. BMP7 and BMP2 have a direct connection with the activation of Runx2 expression [9,46,47], but their action exhibits differences [48]. BMP7 can directly induce the expression of Runx2 but does not regulate the matrix production similarly to BMP2 [48]. It was reported that BMP7 addition increased the activity of ALP in MC3T3 cultures [49]. This observation may imply that inhibition of PACAP receptors has direct connection with BMP7 expression. On the other hand BMP6, the most potent among the BMPs, regulates the osteogenic differentiation and its elevated expression increased the expression of ALP, osterix, osteocalcin and osteopontin [50]. We have already demonstrated that mechanical load of chondrifying cell cultures had a direct connection with PACAP signalling [22]. It induced the expression of collagen type X, a characteristic collagen synthesized by hypertrophic chondrocytes during calcification of cartilage matrix, preceding ossification or establishing the interface of articular cartilage and subchondral bone [27]. It is also known that expression of BMP6 and BMP7 is upregulated during mechanical load in subchondral bone [51]. Our results suggest that BMP6 and BMP7 have partly escaped from the control or fine tuning effects of PACAP signalling pathway and induced the nuclear translocation of Smad1 transcription factor (Figure 6). This pathway also has an influence on ALP activity, which became probably randomly over-activated and generated focal enhancement of mineralization (Figure 6). If these changes occurred without modulation of the activity of any compensatory mechanisms, abnormal bone development and bone morphological disorders would be expected. Nonetheless, the macroscopical characteristics of KO femurs showed only minor alterations. Therefore, it is suggested that BMP over-activation triggers or becomes balanced by another compensatory signalling mechanism. One of these compensatory mechanisms can be the hedgehog signalling, which has a direct connection with PACAP signalisation [27] and also has connection with BMP activation [52]. The activation of PKA induces inhibition of SHH signalling [53], so the absence of PACAP reduces PKA activation, which in turn triggers the over-activation of SHH signalling. The elevated BMP6 and BMP7 expressions in PACAP KO mice may have a direct contact with SHH activation, inducing an approximately normal bone differentiation and formation (Figure 6). Additionally, the expression of IHH was also increased in PACAP KO mice, and it can also be activated by BMPs [54].The expression of IHH can be regulated by BMPs [55] and induces the activation of long bone development. Moreover, Runx2 can have a direct communication with IHH [38]. In chondrogenic cultures we have also shown that the addition of PACAP decreased while mechanical load elevated the expression of IHH [27]. Subsequently, the increased IHH expression may be responsible for compensatory mechanisms, suggesting that PACAP has a negative correlation with IHH activation (Figure 6). Adding together SHH and IHH elevation triggers the action of Gli1, which results in an active bone differentiation process (Figure 6).

In summary, we can conclude that PACAP signalisation is active during long bone development. Moreover, its activation has an important function in the fine tuning of crosstalk between several signalling pathways which are responsible for the formation of anatomically correct femur morphology. These signalling connections keep the appropriate balance of proper secretion of organic and inorganic components, which ensure the mechanical properties of long bones. Therefore, disturbance of PACAP expression implies an altered fragility of cortical bone tissue which may also indicate its possible importance in various bone formation disorders.

## 4. Materials and Methods

### 4.1. Animals

Generation and maintenance of the PACAP-deficient mice on the CD1 background has been described in detail [56,57]. They were backcrossed at least for ten generations with the CD1 strain. Genotype was tested with PCR reactions. For the experiments we sacrificed1-month-old wild-type (WT, *n* = 18, *n* = 10 male, *n* = 8 female) and homozygous PACAP-deficient (PACAP KO, *n* = 18, *n* = 10 male, *n* = 8 female) mice. Animals were fed and watered ad libitum, under light/dark cycles of 12/12 h. All procedures were performed in accordance with the ethical guidelines approved (2 January 2012) by the University of Pécs (permission number: BA02/2000-24/2011).

### 4.2. Whole Limb Alizarin Staining

Hind limbs were removed and only the skin was peeled off. The entire limb was washed in PBS three times and fixed in a 4:1 mixture of absolute ethanol and 40% formaldehyde. After washing in alcohol limbs were incubated in 30, 60 and 80 mM KOH solution for 3 days, 2 days and 1 day, respectively, till the tissues became completely glossy. Residual KOH was removed by washing in PBS three times and samples were stained with alizarin red (Sigma-Aldrich, Saint Louis, MO, USA) for 15 min. Limbs were washed in the concentrated glycerine to remove unbound staining. Photos were made by an Olympus camera, scale was used to demonstrate size differences of hind limbs. Optical density of 1 cm of distal part of femurs was measured by using ImageJ 1.40g freeware and results were normalised to the optical density of wild type samples.

### 4.3. Staining Procedure of Calcificated Tissue

Femurs were dissected and additional tissues were removed. Samples were immediately fixed in 4% paraformaldehyde dissolved in 0.1 M phosphate buffer (PB) for 24 h at room temperature. Tissue was then washed in 0.1 MPB, and cryoprotected in 10% sucrose for 1 h, 20% sucrose in phosphate-buffered saline (PBS) overnight at 4 °C. For cryostat sectioning, the bones were embedded in tissue freezing medium (Tissue-Tek, OCT Compound, Sakura Finetech, Zoeterwade, The Netherlands), cut in a cryostat (Leica, Nussloch, Germany) using a blade for hard tissues. 20–30 serial sections were made from each femur. Sections were mounted on chrome–alum–gelatin-coated subbed slides. Samples were washed three times in PBS and stained with alizarin red (Sigma-Aldrich) for 5 min to evaluate calcium-rich deposits. Calcium phosphate deposition as sign of biomineralization was followed with von Kossa method (Millipore, Billerica, MA, USA). After washing in PBS, samples were incubated in 1% AgNO_3_ solution for 40 min. Slides were removed and exposed to UV for 10 min. Nitrate solution was removed with washing in PBS three times followed by incubation in 5% Na_2_S_2_O_3_ solution for 10 min. Slides were covered with gum arabic. Photomicrographs were taken using an Olympus DP72 camera on a Nikon Eclipse E800 microscope (Nikon Corporation, Tokyo, Japan). Optical density of photos was measured by using ImageJ 1.40g freeware and results were normalised to the optical density of wild type bone.

### 4.4. Staining Procedure of Decalcified Samples

Femurs were dissected and additional tissues were removed as previously described, washed in PBS three times and fixed in a 4:1 mixture of absolute ethanol and 40% formaldehyde. Bones were decalcified in 4% ethylene diamine tetra-acetic acid (EDTA) for four weeks till bones became soft. Then samples were dehydrated in ascending alcohol row and embedded in paraffin. 5 μm of serial sections were made. After rehydration, hematoxilin eosin (Sigma-Aldrich) and picrosirius red (Sigma-Aldrich) stainings were performed. All of the staining protocols were carried out according to the instructions of manufacturer. Photomicrographs were taken using an Olympus DP72 camera on a Nikon Eclipse E800 microscope (Nikon Corporation). Picrosirius staining was analyzed with the polarization module of Nikon Eclipse E800 microscope. Thickness of lamellas was measured by using ImageJ 1.40g freeware. In 3 independent experiments 10 lamellas diameter were calculated and given in pixel unit. Results of wild type animals were compared with gene deficient mice where differences were given in percentage.

### 4.5. Radiographic Analysis (Micro-CT Scan)

Bruker SkyScan 1176 Micro CT Scanner is an X-ray computed tomography system for non invasive imaging of bone morphological parameters. CT scanner was calibrated 50 kV, 500 µA pitch. Reconstruction of the slides was performed by NRecon (1.6.10.4 version) and CTAn (1.15.4.0 version) (Bruker MicroCT, Kontich, Belgium) was used to evaluate them. Femur CT studies obtained by using this micro-CT. Radiographs were used to analyze femur cortical thickness in ventral and dorsal aspects and femur densitometry of PACAP KO and WT mice was also performed. Density was measured in the region of greater trochanter. Using general anesthesia with 1% Euthasol (0.7 µL/10 g) the total femur length (mm) from the greater trochanter to the medial condyle was measured in 18 KO and 18 WT mice.

### 4.6. Measurement of Alkaline Phosphatase Activity

After dissection of hind limbs, femur diaphysis was removed and opened to remove bone marrow from the sample. Cortical bone was washed in PBS three times and 100 µL Radio Immuno Precipitation Assay (RIPA)-buffer (150 mM sodium chloride; 1.0% NP_4_0, 0.5% sodium deoxycholate; 50 mM Tris, pH 8.0) containing protease inhibitors (Aprotinin (10 µg/mL), 5 mMBenzamidine, Leupeptin (10 µg/mL), Trypsine inhibitor (10 µg/mL), 1 mM PMSF, 5 mM EDTA, 1 mM EGTA, 8 mM Na-Fluoride, 1 mM Na-orthovanadate) was added to each experimental group. Bone tissues were mechanically ground in liquid nitrogen (Cryo-Grinder, Nanotestkft., Budapest, Hungary) and sonicated by pulsing burst for 30 s at 40 A (Cole-Parmer, IL, USA). Protein concentration of samples was measured and equalized with BCA-protein assay (Pierce^™^, Rockford, MA, USA). ALP activity measurement was performed according the protocol of manufacturer. Results were measured on 420 nm (Chameleon, Hidex Ltd., Turku, Finland). Activity of ALP was calculated according to the manufacturer’s protocol. Five independent measurements were used to demonstrate significant differences.

### 4.7. Immunohistochemistry

Femurs were dissected and additional tissues were removed. Briefly, bones were washed in PBS three times and fixed in Saint-Marie’s fixative (99% ethanol and 1% anhydrous acetic acid) for 24 h. The bones were decalcified in 4% EDTA for four weeks until the bones became soft. Then samples were dehydrated in descending alcohol row and embedded in paraffin. 5 μm serial sections were made. Sections were rehydrated in ascending alcohol row and washed in PBST (phosphate buffered saline supplemented with 1% Tween-20) three times. Nonspecific binding sites were blocked in PBST supplemented with 1% bovine serum albumin (BSA, Amresco LLC, Solon, OH, USA) at 37 °C for 30 min.

For collagen type I, Smad1 and Runx2 immunohistochemistry sections were incubated in anti-Collagen type I. (Sigma-Aldrich) at a dilution of 1:500, in anti-Smad1 (Cell Signaling, Danvers, MA, USA) at a dilution of 1:600, and anti-Runx2 (Cell Signaling, Danvers, MA, USA) at a dilution of 1:600 at 4°C overnight. Primary antibodies were visualised with anti-rabbit Alexa555 and/or anti-mouse Alexa488 secondary antibodies (Life Technologies Corporation, Carlsbad, CA, USA) at a dilution of 1:1000. Slides were mounted in Vectashield Hard Set mounting medium (Vector Laboratories, Peers below signals represent integrateterborough, UK) containing DAPI to visualise the cell nuclei. Photomicrographs of collagen type I were taken using an Olympus DP72 camera on a Nikon Eclipse E800 microscope (Nikon Corporation, Tokyo, Japan). Images were acquired using cellSense Entry 1.5 software (Olympus, Shinjuku, Tokyo, Japan) with constant camera settings to allow comparison of fluorescent signal intensities. For investigation of subcellular localization of Smad1 and Runx2 fluorescent images were taken with an Olympus FV1000S confocal microscope (Olympus Co., Tokyo, Japan) using 40× oil immersion objective (NA: 1.3). For excitation, laser lines of 543 nm and 488 nm were used. The average pixel time was 4 µs. Z image series of 1 µm optical thickness were recorded in sequential scan mode. Images of Alexa555 and DAPI were overlaid using Adobe Photoshop version 10.0 software.

### 4.8. RT-PCR Analysis

In liquid nitrogen femurs were cryo-ground and collected in TriReagent (Applied Biosystems, Foster City, CA, USA). 20% RNase free chloroform was added to the dissolved samples and followed by a centrifugation at 4 °C at 10,000×*g* for 15 min. Samples were incubated in 500 µL of RNase-free isopropanol at −20 °C for 1 h then total RNA was harvested in RNase free water and stored at −20 °C. For reverse transcriptase reaction the assays contained 2 µg RNA, 0.112 µM oligo(dT), 0.5 mM deoxynucleotide triphosphate (dNTP), 200 units of High Capacity RT (Applied Bio-Systems, Waltham, MA, USA) in 1× RT buffer and RT reaction was run in a thermal cycler (Labnet MultiGene™ 96-well Gradient Thermal Cycler; Labnet International, Edison, NJ, USA). Sequences of primer pairs, amplimer sizes and further details are given in Table 1. Amplifications were performed in a thermal cycler (Labnet MultiGene™ 96-well Gradient Thermal Cycler; Labnet International). Final volume of assay mixture was 21 μL (containing 1 μL forward and reverse primers [0.4 μM], 0.5 μL dNTP [200 μM], and 5 units of Promega GoTaq^®^ DNA polymerase in 1× reaction buffer) and protocols were set as follows: 95 °C, 2 min, followed by 35 cycles (denaturation, 94 °C, 1 min; annealing at optimised temperatures as given in Table 1 for 1 min; extension, 72 °C, 90 s) and then 72 °C, for 10 min. Amplimers were analysed in 1.2% agarose gel containing ethidium bromide. Actin was used as internal control. Optical density of signals was measured by using ImageJ 1.40 g freeware and results were normalised to the optical density of control tissue.

### 4.9. Western Blot Analysis

Tissues were cryo-grinded in liquid nitrogen. After centrifugation, tissue pellets were suspended in 100 μL of homogenization RIPA. Samples were stored at −70 °C. Suspensions were sonicated by pulsing burst for 30 s at 40 A (Cole-Parmer, Vernon Hills, IL, USA). For Western blotting, total cell lysates were used. Samples for SDS-PAGE were prepared by the addition of Laemmli electrophoresis sample buffer (4% SDS, 10% 2-mercaptoehtanol, 20% glycerol, 0.004% bromophenol blue, 0.125 M Tris HCl pH 6.8) to cell lysates to set equal protein concentration of samples, and boiled for 10 min. About 40 µg of protein was separated by 7.5% SDS-PAGE gel for detection of PAC1, VPAC1, VPAC2, PKA, ALP, osteocalcin, osteonectin, osterix, CREB, P-CREB, collagen type I, Runx2, BMP2, BMP4, BMP6, BMP7, BMPR1, Smad1, SHH, IHH, PTCH1 and Gli1. Proteins were transferred electrophoretically to nitrocellulose membranes. After blocking with 5% non-fat dry milk in phosphate buffered saline (PBST) with 0.1% Tween 20, membranes were washed and exposed to the primary antibodies overnight at 4 °C in the dilution as given in Table 2. After washing for 30 min in PBST, membranes were incubated with anti-rabbit IgG (Bio-Rad Laboratories, Hercules, CA, USA) in 1:1500, anti-goat IgG (Sigma-Aldrich) in 1:2000 and anti-mouse IgG (Bio-Rad Laboratories) in 1:1500 dilution. Signals were detected by enhanced chemiluminescence (Pierce^TM^) according to the instructions of the manufacturer. Signals were developed on X-ray films and documented by gel documentary system (Fluorchem E, ProteinSimple, Santa Clara, CA, USA). For negative or positive controls to test antibodies brain, testis and cartilage were used from WT animals (Figures S4D, S5E and S6D). Optical density of Western blot signals was measured by using ImageJ 1.40g freeware and results were normalised to that of control samples.

### 4.10. Fracture Test

Three point bending tests were performed on mouse femurs using a Chatillon TCD225 tensile test machine. The center part of the bones was loaded by a 5 mm diameter cylindrical head (Figure S1A). Velocity of the head was 2 mm/min during the experiments. Value of the loading force at crack (F_m_) as well as the value of the bending deformation at crack (Δl_m_) were evaluated from the deformation-force diagrams (Figure S1B).

### 4.11. Statistical Analysis

All data are representative of at least three different experiments. Where applicable, data are expressed as mean ± SEM. Statistical analysis was performed by Student’s *t* test. Threshold for statistically significant differences as compared to respective control (wild type animals) was set at * *p* < 0.05.

## Figures and Tables

**Table 1:** Nucleotide sequences, amplification sites, GenBank accession numbers, amplimer sizes and PCR reaction conditions for each primer pair are shown.

Gene	Primer	Nucleotide Sequence (5′→3′)	GenBank ID	Annealing Temperature	Amplimer Size (bp)
***Alkaline phosphatase*** ***(Alpl)***	sense	GAA GTC CGT GGG CAT CGT (474–491)	NM013059	59 °C	347
	antisense	CAG TGC GGT TCC AGA CAT AG (801–820)			
***BMP2*** ***(Bmp2)***	sense	AAG CCA GGT GTC TCC AAG (697–714)	NM017178.1	53 °C	209
	antisense	AAG TCC ACA TAC AAA GGG TG (886–905)			
***BMP4*** ***(Bmp4)***	sense	TAG TCC CAA GCA TCA CCC (876–893)	NM012827.2	53 °C	294
	antisense	TCG TAC TCG TCC AGA TAC AAC (1149–1169)			
***BMP6*** ***(Bmp6)***	sense	CCC AGA TTC CTG AGG GTG A (936–954)	NM013107.1	56 °C	248
	antisense	CAT GTT GTG CTG CGG TGT (1166–1183)			
***BMP7*** ***(Bmp7)***	sense	AGG GAG TCC GAC CTC TTC T (607–625)	NM001191856.1	54 °C	297
	antisense	GTT CTG GCT GCG TTG TTT (886–903)			
***BMPR1*** ***(Bmpr1a)***	sense	CCA TTG CTT TGC CAT TAT (240–257)	NM009758.4	47 °C	487
	antisense	TTT ACC AAC CTG CCG AAC (709–726)			
***Collagen type I*** ***(Col1a1)***	sense	GGG CGA GTG CTG TGC TTT (348–365)	NM007742.3	60 °C	388
	antisense	GGG ACC CAT TGG ACC TGA A (717–735)			
***CREB*** ***(Creb1)***	sense	AGA TTG CCA CAT TAG CCC (95–112)	NM031017.1	52 °C	441
	antisense	GCT GTA TTG CTC CTC CCT (518–535)			
***Actin*** ***(Actb)***	sense	GCC AAC CGT GAA AAG ATG A (419–437)	NM007393.5	54 °C	462
	antisense	CAA GAA GGA AGG CTG GAA AA (861–880)			
***Gli1*** ***(Gli1)***	sense	CCA CCC TAC CTC TGT CTA TTC G (2201–2222)	NM010296.2	49 °C	423
	antisense	CAC CCT TGT TCT GGT TTT ACC (2603–2623)			
***IHH*** ***(Ihh)***	sense	CCA ACT ACA ATC CCG ACA TCA (248–268)	NM053384.1	58 °C	477
	antisense	GTC TTC ATC CCA GCC TTC C (390–408)			
***Osterix*** ***(Sp7)***	sense	GCC TAC TTA CCC GTC TGA CTT T (525–543)	NM001037632.1	56 °C	131
	antisense	GCC CAC TAT TGC CAA CTG C (634–652)			
***Osteocalcin*** ***(Bglap2)***	sense	TAA GGT GGT GAA TAG ACT CCG (123–143)	NM013414.1	56 °C	143
	antisense	CCT GGA AGC CAA TGT GGT (248–265)			
***PAC1*** ***(ADCYAP1R1)***	sense	CTA CGC CCT TTA CTA CCC AG (210–229)	NM016989.2	49 °C	247
	antisense	GTA TTT CTT GAC AGC CAT TTG T (435–456)			
***PKA*** ***(Prkaca)***	sense	GCA AAG GCT ACA ACA AGG C (847–865)	NM008854	53 °C	280
	antisense	ATG GCA ATC CAG TCA ATC G (1109–1126)			
***Osteopontin*** ***(Spp1)***	sense	GCT GAA GCC TGA CCC ATC T (126–144)	X51834	59 °C	494
	antisense	TCC CGT TGC TGT CCT GAT (602–619)			
***PTCH1*** ***(Ptch1)***	sense	GGA ACT TAT CAC GGA GAC AG (579–578)	NM053566.1	56 °C	368
	antisense	AAC CTT GAC ATC CAC CAT T (928–946)			
***Runx2*** ***(Runx2)***	sense	GGA CGA GGC AAG AGT TTC A (598–616)	NM001278483.1	55 °C	249
	antisense	TGG TGC AGA GTT CAG GGA G (828–846)			
***SHH*** ***(Shh)***	sense	TCG TGC TAC GCA GTC ATC G (1042–1060)	NM017221.1	56 °C	156
	antisense	CCT CGC TTC CGC TAC AGA (1180–1197)			
***Smad1*** ***(Smad1)***	sense	AGC ACC TAC CCT CAC TCC C (935–953)	NM013130.2	56 °C	306
	antisense	GAA ACC ATC CAC CAA CAC G (1222–1240)			
***VPAC1*** ***(VIPR1)***	sense	GTT CTA TGG CAC GGT CAA (376–393)	NM001097523	52 °C	216
	antisense	AGC AAT GTT CGG GTT CTC (573–590)			
***VPAC2*** ***(VIPR2)***	sense	TCG GAA CTA CAT CCA TCT (477–497)	NM001014970	48 °C	177
	antisense	TTT GCC ATA ACA CCA TAC (636–653)			

**Table 2:** Tables of antibodies used in the experiments.

Antibody	Host Animal	Dilution	Distributor
Anti-PAC1	rabbit, polyclonal,	1:600	Sigma-Aldrich, St. Louis, MO, USA
Anti-VPAC1	rabbit, polyclonal,	1:800	Alomone Labs., Jerusalem, Israel
Anti-VPAC2	rabbit, polyclonal,	1:600	Abcam, Camridge, UK
Anti-Coll. I.	mouse, monoclonal,	1:1000	Sigma-Aldrich, St. Louis, MO, USA
Anti-CREB	rabbit, polyclonal,	1:800	Millipore, Billerica, MA, USA
Anti-P-CREB	rabbit, polyclonal,	1:800	Millipore, Billerica, MA, USA
Anti-Osterix	rabbit, polyclonal,	1:200	SantaCruz Biotechnology Inc., Santa Cruz, CA, USA
Anti-Osteocalcin	rabbit, polyclonal,	1:600	Abcam, Camridge, UK
Anti-Osteopontin	rabbit, polyclonal,	1:500	Abcam, Camridge, UK
Anti-ALP	rabbit, polyclonal,	1:500	Abcam, Camridge, UK
Anti-SHH	rabbit, polyclonal,	1:600	Cell Signaling, Danvers, MA, USA
Anti-IHH	rabbit, polyclonal,	1:600	Millipore, Billerica, MA, USA
Anti-PKA	rabbit, polyclonal,	1:800	Cell Signaling, Danvers, MA, USA
Anti-Runx2	rabbit, polyclonal,	1:500	Cell Signaling, Danvers, MA, USA
Anti-Gli1	rabbit, polyclonal,	1:600	Cell Signaling, Danvers, MA, USA
Anti-BMP2	mouse, monoclonal,	1:500	Abcam, Camridge, UK
Anti-BMP4	rabbit, polyclonal,	1:600	Cell Signaling, Danvers, MA, USA
Anti-BMP6	rabbit, polyclonal,	1:200	SantaCruz Biotechnology Inc., Santa Cruz, CA, USA
Anti-BMP7	rabbit, polyclonal,	1:600	Abcam, Camridge, UK
Anti-BMPR1	mouse, monoclonal,	1:600	Abcam, Camridge, UK
Anti-Smad1	rabbit, polyclonal,	1:600	Cell Signaling, Danvers, MA, USA
Anti-PTCH1	rabbit, polyclonal,	1:800	Cell Signaling, Danvers, MA, USA
Anti-Actin	mouse, monoclonal,	1:10,000	Sigma-Aldrich, St. Louis, MO, USA

## Supplementary Materials

Supplementary materials can be found at http://www.mdpi.com/1422-0067/19/9/2538/s1.

## Abbreviations

ALP	Alkaline phosphatase
BMP	Bone morphogenetic protein
cAMP	Cyclic adenosine monophosphate
CNS	Central nervous system
CREB	cAMP response element-binding protein
DMEM	Dulbecco’s Modified Eagle’s Medium
dNTP	deoxynucleotide triphosphate
ECM	Extracellular matrix
EDTA	Ethylene diamine tetra-acetic acid
EGTA	Ethylene glycol-bis(β-aminoethyl ether)-*N*,*N*,*N*′,*N*′-tetra acetic acid
FBS	Foetal bovine serum
FGF	Fibroblast growth factor; Gli, glioma-associated oncogene
HH	hedgehog
HEPES	4-(2-hydroxyethyl)-1-piperazine ethanesulfonic acid
hMSC	human mesenchymal stem cell
IHH	Indian Hedgehog
MAPK	Mitogen-activated protein kinase
PAC1	Pituitary adenylate cyclase-activating polypeptide type I receptor
PACAP	Pituitary adenylate cyclase activating polypeptide, phosphate buffered saline
PBST	Phosphate buffered saline supplemented with 1% Tween-20
PLC	Phospholipase C
PKA	Protein kinase A
PKC	Protein kinase C
PTCH	Patched
RT-PCR	Reverse transcription followed by polymerase chain reaction Runt-related transcription factor 2
SHH	Sonic Hedgehog
TGFβ	Transforming growth factor-β
VIP	Vasoactive intestinal polypeptide
VPAC	Vasoactive intestinal polypeptide receptor
WNT	Wingless int1

## References

[1] Majidinia M., Sadeghpour A., Yousefi B. (2017). The roles of signaling pathways in bone repair and regeneration. J. Cell. Physiol..

[2] Oftadeh R., Perez-Viloria M., Villa-Camacho J.C., Vaziri A., Nazarian A. (2015). Biomechanics and mechanobiology of trabecular bone: A review. J. Biomech. Eng..

[3] Hegde V., Jo J.E., Andreopoulou P., Lane J.M. (2016). Effect of osteoporosis medications on fracture healing. Osteoporos. Int..

[4] Wu M., Chen G., Li Y.P. (2016). TGF-beta and BMP signaling in osteoblast, skeletal development, and bone formation, homeostasis and disease. Bone Res..

[5] Forlino A., Marini J.C. (2016). Osteogenesis imperfecta. Lancet.

[6] Ascenzi M.G., Roe A.K. (2012). The osteon: The micromechanical unit of compact bone. Front. Biosci..

[7] Shahi M., Peymani A., Sahmani M. (2017). Regulation of Bone Metabolism. Rep. Biochem. Mol. Biol..

[8] Zhou W., Yu L., Fan J., Wan B., Jiang T., Yin J., Huang Y., Li Q., Yin G., Hu Z. (2017). Endogenous Parathyroid Hormone Promotes Fracture Healing by Increasing Expression of BMPR2 through cAMP/PKA/CREB Pathway in Mice. Cell. Physiol. Biochem..

[9] Li Z., Wang W., Xu H., Ning Y., Fang W., Liao W., Zou J., Yang Y., Shao N. (2017). Effects of altered CXCL12/CXCR4 axis on BMP2/Smad/Runx2/Osterix axis and osteogenic gene expressions during osteogenic differentiation of MSCs. Am. J. Transl. Res..

[10] Vaudry D., Falluel-Morel A., Bourgault S., Basille M., Burel D., Wurtz O., Fournier A., Chow B.K., Hashimoto H., Galas L. (2009). Pituitary adenylate cyclase-activating polypeptide and its receptors: 20 years after the discovery. Pharmacol. Rev..

[11] Miyata A., Arimura A., Dahl R.R., Minamino N., Uehara A., Jiang L., Culler M.D., Coy D.H. (1989). Isolation of a novel 38 residue-hypothalamic polypeptide which stimulates adenylate cyclase in pituitary cells. Biochem. Biophys. Res. Commun..

[12] Barberi M., Di Paolo V., Latini S., Guglielmo M.C., Cecconi S., Canipari R. (2013). Expression and functional activity of PACAP and its receptors on cumulus cells: Effects on oocyte maturation. Mol. Cell. Endocrinol..

[13] Heimesaat M.M., Dunay I.R., Schulze S., Fischer A., Grundmann U., Alutis M., Kuhl A.A., Tamas A., Toth G., Dunay M.P. (2014). Pituitary adenylate cyclase-activating polypeptide ameliorates experimental acute ileitis and extra-intestinal sequelae. PLoS ONE.

[14] Banki E., Kovacs K., Nagy D., Juhasz T., Degrell P., Csanaky K., Kiss P., Jancso G., Toth G., Tamas A. (2014). Molecular mechanisms underlying the Nephroprotective effects of PACAP in diabetes. J. Mol. Neurosci..

[15] Csanaky K., Banki E., Szabadfi K., Reglodi D., Tarcai I., Czegledi L., Helyes Z., Ertl T., Gyarmati J., Szanto Z. (2012). Changes in PACAP immunoreactivity in human milk and presence of PAC1 receptor in mammary gland during lactation. J. Mol. Neurosci..

[16] Juhasz T., Matta C., Katona E., Somogyi C., Takacs R., Gergely P., Csernoch L., Panyi G., Toth G., Reglodi D. (2014). Pituitary adenylate cyclase activating polypeptide (PACAP) signalling exerts chondrogenesis promoting and protecting effects: Implication of calcineurin as a downstream target. PLoS ONE.

[17] Mester L., Kovacs K., Racz B., Solti I., Atlasz T., Szabadfi K., Tamas A., Reglodi D. (2011). Pituitary adenylate cyclase-activating polypeptide is protective against oxidative stress in human retinal pigment epithelial cells. J. Mol. Neurosci..

[18] Laszlo E., Varga A., Kovacs K., Jancso G., Kiss P., Tamas A., Szakaly P., Fulop B., Reglodi D. (2015). Ischemia/reperfusion-induced Kidney Injury in Heterozygous PACAP-deficient Mice. Transplant. Proc..

[19] Gomariz R.P., Juarranz Y., Abad C., Arranz A., Leceta J., Martinez C. (2006). VIP-PACAP system in immunity: New insights for multitarget therapy. Ann. N. Y. Acad. Sci..

[20] Tsuchida M., Nakamachi T., Sugiyama K., Tsuchikawa D., Watanabe J., Hori M., Yoshikawa A., Imai N., Kagami N., Matkovits A. (2014). PACAP stimulates functional recovery after spinal cord injury through axonal regeneration. J. Mol. Neurosci..

[21] Nakamura K., Nakamachi T., Endo K., Ito K., Machida T., Oka T., Hori M., Ishizaka K., Shioda S. (2014). Distribution of pituitary adenylate cyclase-activating polypeptide (PACAP) in the human testis and in testicular germ cell tumors. Andrologia.

[22] Juhasz T., Helgadottir S.L., Tamas A., Reglodi D., Zakany R. (2015). PACAP and VIP signaling in chondrogenesis and osteogenesis. Peptides.

[23] Lundberg P., Lundgren I., Mukohyama H., Lehenkari P.P., Horton M.A., Lerner U.H. (2001). Vasoactive intestinal peptide (VIP)/pituitary adenylate cyclase-activating peptide receptor subtypes in mouse calvarial osteoblasts: Presence of VIP-2 receptors and differentiation-induced expression of VIP-1 receptors. Endocrinology.

[24] Juhasz T., Matta C., Katona E., Somogyi C., Takacs R., Hajdu T., Helgadottir S.L., Fodor J., Csernoch L., Toth G. (2014). Pituitary adenylate cyclase-activating polypeptide (PACAP) signalling enhances osteogenesis in UMR-106 cell line. J. Mol. Neurosci..

[25] Nagata A., Tanaka T., Minezawa A., Poyurovsky M., Mayama T., Suzuki S., Hashimoto N., Yoshida T., Suyama K., Miyata A. (2009). cAMP activation by PACAP/VIP stimulates IL-6 release and inhibits osteoblastic differentiation through VPAC2 receptor in osteoblastic MC3T3 cells. J. Cell. Physiol..

[26] Strange-Vognsen H.H., Arnbjerg J., Hannibal J. (1997). Immunocytochemical demonstration of pituitary adenylate cyclase activating polypeptide (PACAP) in the porcine epiphyseal cartilage canals. Neuropeptides.

[27] Juhasz T., Szentleleky E., Somogyi C.S., Takacs R., Dobrosi N., Engler M., Tamas A., Reglodi D., Zakany R. (2015). Pituitary Adenylate Cyclase Activating Polypeptide (PACAP) Pathway Is Induced by Mechanical Load and Reduces the Activity of Hedgehog Signaling in Chondrogenic Micromass Cell Cultures. Int. J. Mol. Sci..

[28] May V., Clason T.A., Buttolph T.R., Girard B.M., Parsons R.L. (2014). Calcium influx, but not intracellular calcium release, supports PACAP-mediated ERK activation in HEK PAC1 receptor cells. J. Mol. Neurosci..

[29] Watanabe J., Nakamachi T., Matsuno R., Hayashi D., Nakamura M., Kikuyama S., Nakajo S., Shioda S. (2007). Localization, characterization and function of pituitary adenylate cyclase-activating polypeptide during brain development. Peptides.

[30] Toriyama M., Mizuno N., Fukami T., Iguchi T., Toriyama M., Tago K., Itoh H. (2012). Phosphorylation of doublecortin by protein kinase A orchestrates microtubule and actin dynamics to promote neuronal progenitor cell migration. J. Biol. Chem..

[31] Farkas J., Sandor B., Tamas A., Kiss P., Hashimoto H., Nagy A.D., Fulop B.D., Juhasz T., Manavalan S., Reglodi D. (2017). Early Neurobehavioral Development of Mice Lacking Endogenous PACAP. J. Mol. Neurosci..

[32] Baumgartner R., Heeren N., Quast D., Babst R., Brunner A. (2015). Is the cortical thickness index a valid parameter to assess bone mineral density in geriatric patients with hip fractures?. Arch. Orthop. Trauma Surg..

[33] Alam I., Alkhouli M., Gerard-O’Riley R.L., Wright W.B., Acton D., Gray A.K., Patel B., Reilly A.M., Lim K.E., Robling A.G. (2016). Osteoblast-Specific Overexpression of Human WNT16 Increases Both Cortical and Trabecular Bone Mass and Structure in Mice. Endocrinology.

[34] Sinha P., Aarnisalo P., Chubb R., Poulton I.J., Guo J., Nachtrab G., Kimura T., Swami S., Saeed H., Chen M. (2016). Loss of Gsalpha in the Postnatal Skeleton Leads to Low Bone Mass and a Blunted Response to Anabolic Parathyroid Hormone Therapy. J. Biol. Chem..

[35] Zakany R., Bako E., Felszeghy S., Hollo K., Balazs M., Bardos H., Gergely P., Modis L. (2001). Okadaic acid-induced inhibition of protein phosphatase 2A enhances chondrogenesis in chicken limb bud micromass cell cultures. Anat. Embryol..

[36] Zakany R., Szucs K., Bako E., Felszeghy S., Czifra G., Biro T., Modis L., Gergely P. (2002). Protein phosphatase 2A is involved in the regulation of protein kinase A signaling pathway during in vitro chondrogenesis. Exp. Cell Res..

[37] Zhang H., Li L., Dong Q., Wang Y., Feng Q., Ou X., Zhou P., He T., Luo J. (2015). Activation of PKA/CREB Signaling is Involved in BMP9-Induced Osteogenic Differentiation of Mesenchymal Stem Cells. Cell. Physiol. Biochem..

[38] Laurie L.E., Kokubo H., Nakamura M., Saga Y., Funato N. (2016). The Transcription Factor Hand1 Is Involved in Runx2-Ihh-Regulated Endochondral Ossification. PLoS ONE.

[39] Poole K.E., Treece G.M., Mayhew P.M., Vaculik J., Dungl P., Horak M., Stepan J.J., Gee A.H. (2012). Cortical thickness mapping to identify focal osteoporosis in patients with hip fracture. PLoS ONE.

[40] Sandor B., Fintor K., Felszeghy S., Juhasz T., Reglodi D., Mark L., Kiss P., Jungling A., Fulop B.D., Nagy A.D. (2014). Structural and morphometric comparison of the molar teeth in pre-eruptive developmental stage of PACAP-deficient and wild-type mice. J. Mol. Neurosci..

[41] Sharma U., Pal D., Prasad R. (2014). Alkaline phosphatase: An overview. IndianJ. Clin. Biochem..

[42] Schaffler M.B., Choi K., Milgrom C. (1995). Aging and matrix microdamage accumulation in human compact bone. Bone.

[43] Hu L., Su P., Yin C., Zhang Y., Li R., Yan K., Chen Z., Li D., Zhang G., Wang L. (2017). Microtubule actin crosslinking factor 1 promotes osteoblast differentiation by promoting beta-catenin/TCF1/Runx2 signaling axis. J. Cell. Physiol..

[44] Shen B., Mu J.X., Pei F.X. (2007). Relationship among bone mineral density, collagen composition, and biomechanical properties of callus in the healing of osteoporotic fracture. Chin. J. Traumatol..

[45] Caetano-Lopes J., Nery A.M., Canhao H., Duarte J., Cascao R., Rodrigues A., Perpetuo I.P., Abdulghani S., Amaral P.M., Sakaguchi S. (2010). Chronic arthritis leads to disturbances in the bone collagen network. Arthritis Res. Ther..

[46] Tou L., Quibria N., Alexander J.M. (2003). Transcriptional regulation of the human Runx2/Cbfa1 gene promoter by bone morphogenetic protein-7. Mol. Cell. Endocrinol..

[47] Li J., Hao L., Wu J., Zhang J., Su J. (2016). Linarin promotes osteogenic differentiation by activating the BMP-2/RUNX2 pathway via protein kinase A signaling. Int. J. Mol. Med..

[48] Bais M.V., Wigner N., Young M., Toholka R., Graves D.T., Morgan E.F., Gerstenfeld L.C., Einhorn T.A. (2009). BMP2 is essential for post natal osteogenesis but not for recruitment of osteogenic stem cells. Bone.

[49] Hadjicharalambous C., Kozlova D., Sokolova V., Epple M., Chatzinikolaidou M. (2015). Calcium phosphate nanoparticles carrying BMP-7 plasmid DNA induce an osteogenic response in MC3T3-E1 pre-osteoblasts. J. Biomed. Mater. Res..

[50] Friedman M.S., Long M.W., Hankenson K.D. (2006). Osteogenic differentiation of human mesenchymal stem cells is regulated by bone morphogenetic protein-6. J. Cell. Biochem..

[51] Nam J., Perera P., Rath B., Agarwal S. (2013). Dynamic regulation of bone morphogenetic proteins in engineered osteochondral constructs by biomechanical stimulation. Tissue Eng..

[52] Jiang Q., Du J., Yin X., Shan Z., Ma Y., Ma P., Du J., Fan Z. (2013). Shh signaling, negatively regulated by BMP signaling, inhibits the osteo/dentinogenic differentiation potentials of mesenchymal stem cells from apical papilla. Mol. Cell. Biochem..

[53] Niewiadomski P., Zhujiang A., Youssef M., Waschek J.A. (2013). Interaction of PACAP with Sonic hedgehog reveals complex regulation of the hedgehog pathway by PKA. Cell. Signal..

[54] Seki K., Hata A. (2004). Indian hedgehog gene is a target of the bone morphogenetic protein signaling pathway. J. Biol. Chem..

[55] Duench K., Franz-Odendaal T.A. (2012). BMP and Hedgehog signaling during the development of scleral ossicles. Dev. Biol..

[56] Hashimoto H., Shintani N., Tanaka K., Mori W., Hirose M., Matsuda T., Sakaue M., Miyazaki J., Niwa H., Tashiro F. (2001). Altered psychomotor behaviors in mice lacking pituitary adenylate cyclase-activating polypeptide (PACAP). Proc. Natl. Acad. Sci. USA.

[57] Hashimoto H., Hashimoto R., Shintani N., Tanaka K., Yamamoto A., Hatanaka M., Guo X., Morita Y., Tanida M., Nagai K. (2009). Depression-like behavior in the forced swimming test in PACAP-deficient mice: Amelioration by the atypical antipsychotic risperidone. J. Neurochem..

