# Supplementary material for: Signalling Alterations in Bones of Pituitary Adenylate Cyclase Activating Polypeptide (PACAP) Gene Deficient Mice
Source: Int J Mol Sci. 2018 Aug 27;19(9):2538. doi: 10.3390/ijms19092538 (PMC6163297; doi:10.3390/ijms19092538)
Supplement: Supplementary file 1 [file ijms-19-02538-s001.pdf]

Supplementary Figures

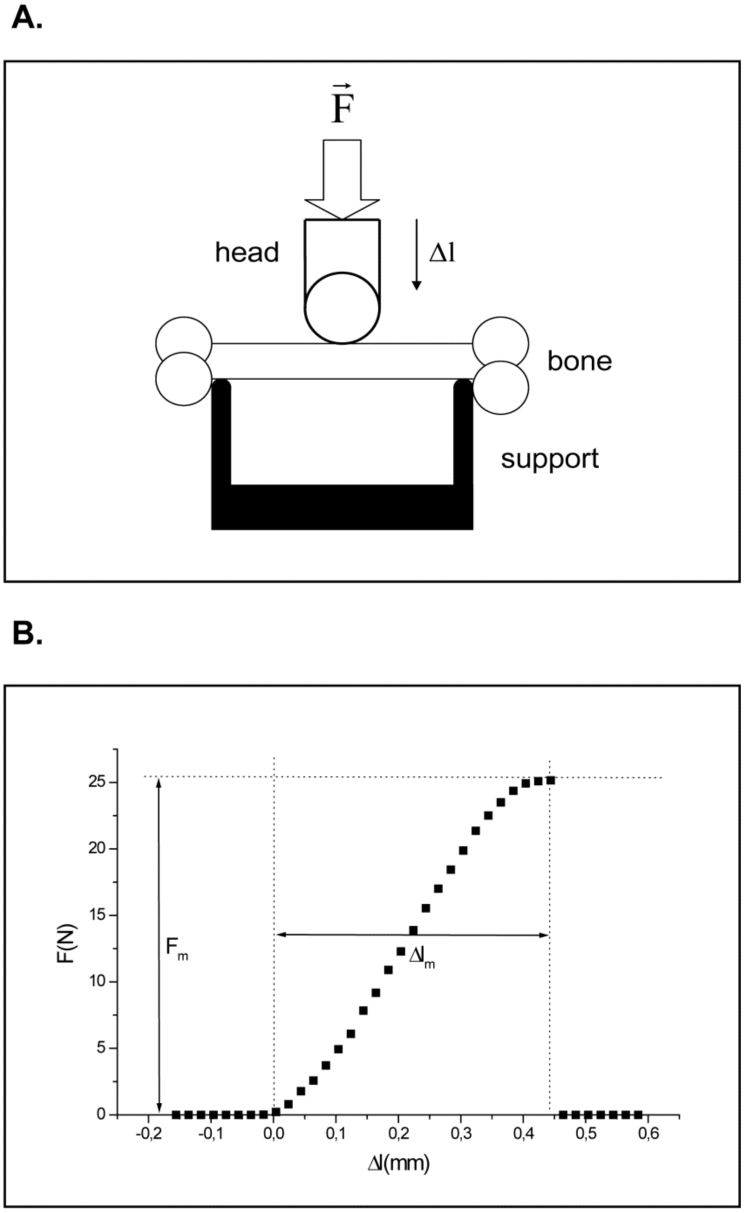

Figure S1. Schematic figure of the three point bending test machine (**A**). The specimen was loaded in the middle by a 5 mm cilindrical test tool. The velocity of the loading head was 2 mm/min during the bending test. (**B**) A typical experimental loading force (F) - bending deformation (Δl) curve. The value of the loading force at crack (Fm) as well as the value of the bending deformation at crack (Δlm) is indicated. These data were evaluated from this type of diagrams.

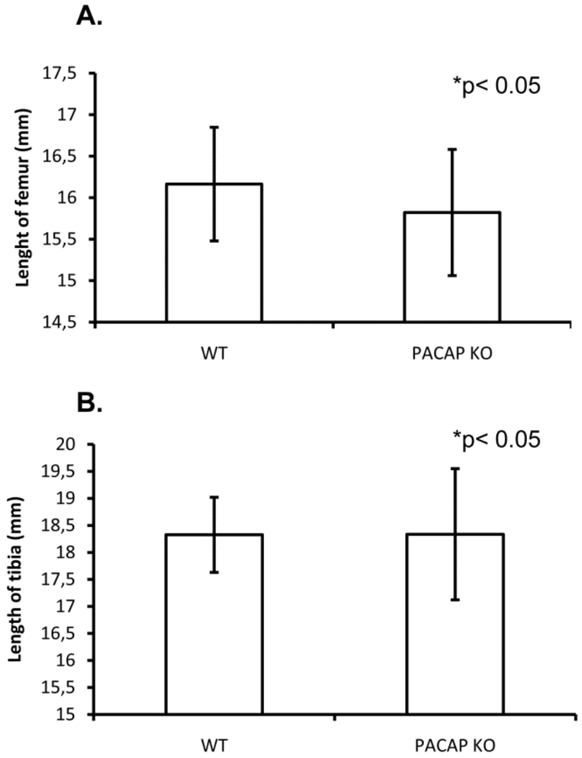

Figure S2. The average length of the femur (**A**) and tibia (**B**) WT and PACAP KO mice were shown no significant differences. The measurement was performed on/in the CT scan.

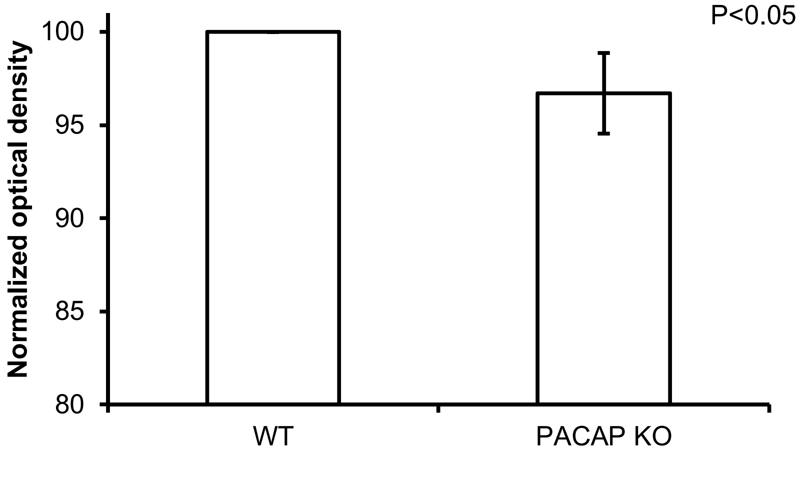

Figure S3. Statistical analysis of whole limb alizarin staining of Figure 1 A. All data presented are the averages of at least 5 independent experiments. Optical density of limbs was measured by using ImageJ 1.40g freeware and results were normalised to the optical density of WT limb. Statistical analysis was performed by Student's *t*-test.

A. Densitometry of von Kossa and alizarin stainings

von Kossa staining proximal diaphysis

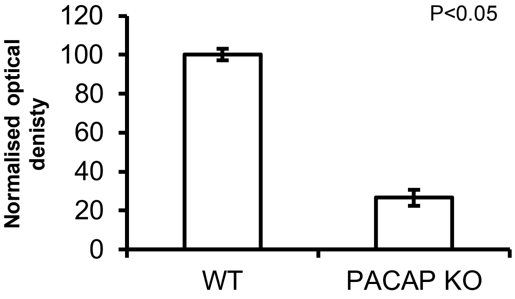

von Kossa staining distal diaphysis

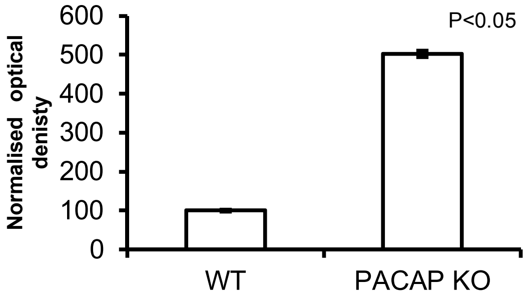

Alizarin red staining proximal diaphysis

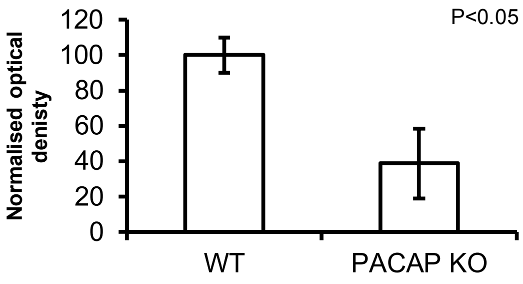

Alizarin red staining distal diaphysis

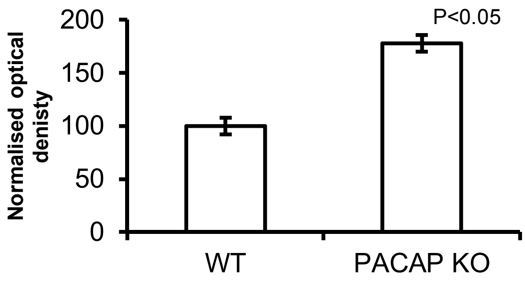

B. Densitometry of RT-PCR reactions

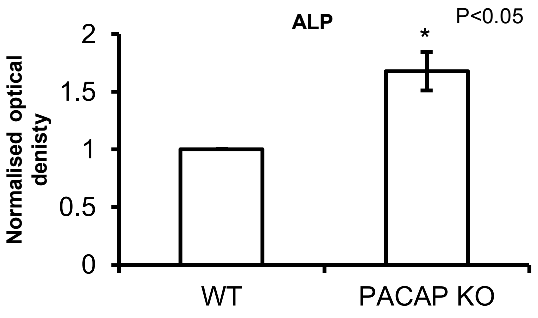

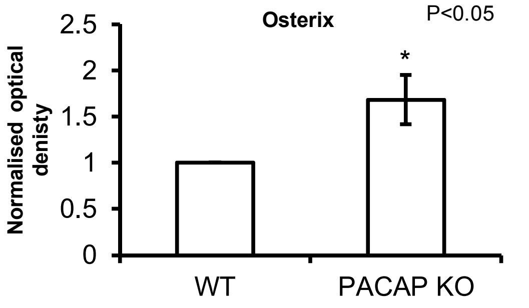

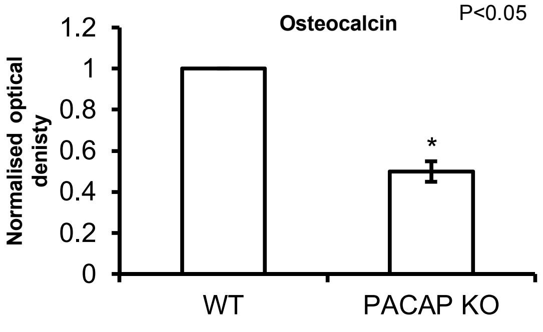

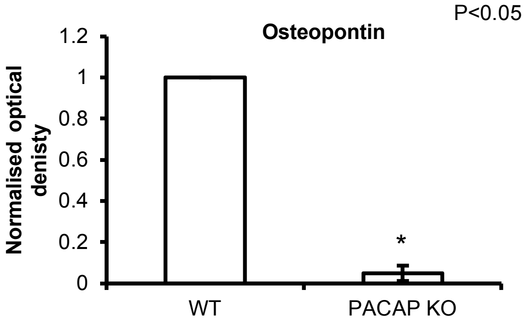

C. Densitometry of Western blot reactions

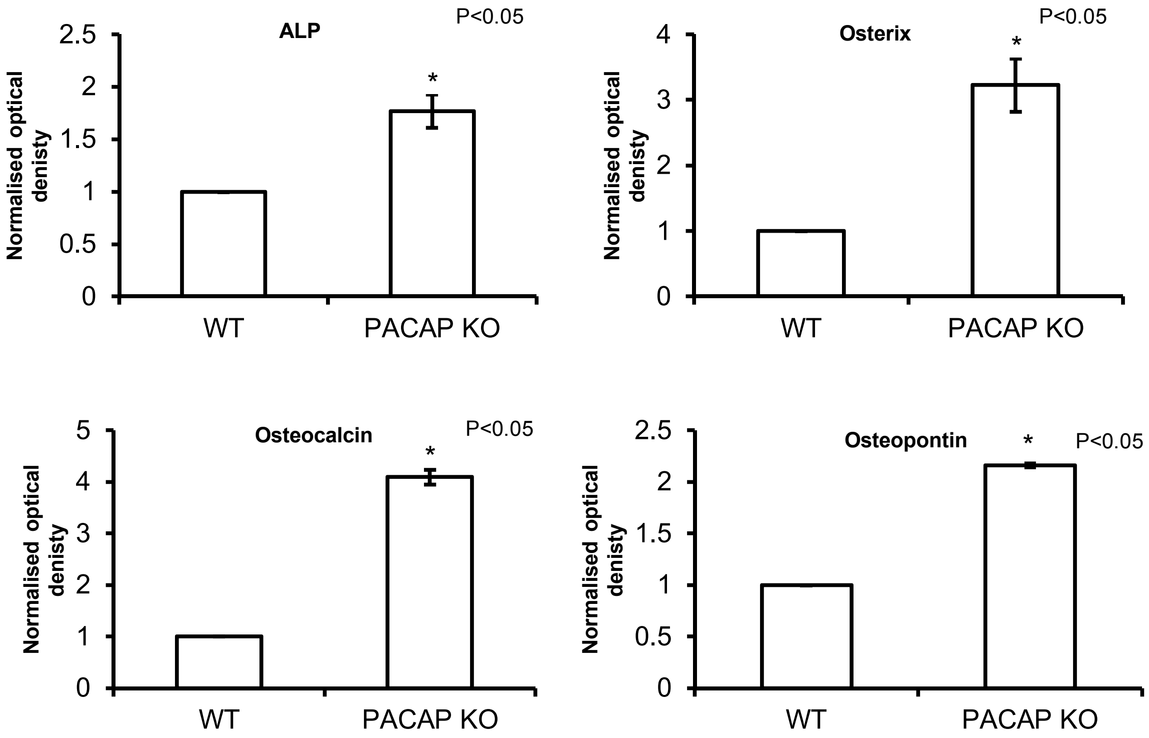

D. Negative control for ALP Western blot reaction

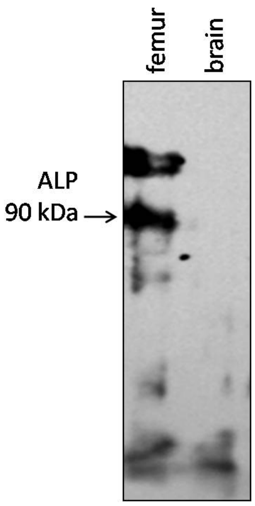

Figure S4. (**A**) Statistical analysis of von Kossa and alizarin stainings of Figure 2 A and B. Optical density of limbs was measured and results were normalised to the optical density of WT limb. (**B**) and (**C**) Statistical analysis of RT-PCR and Western blot data of Figure 2 C and D. Optical density of limbs was measured by using ImageJ 1.40g freeware. All data are the average of at least three different experiments. Statistical analysis was performed by Student's *t*-test. All data were normalized on Actin and data are expressed as mean ± SEM. Asterisks indicate significant (*P<0.05) alteration of expression as compared to the respective control.

(**D**) For negative control of ALP Western blot reaction brain of WT mouse was used. (WT, wild type; PACAP KO).

A. Relative thickness of collagen lamellae

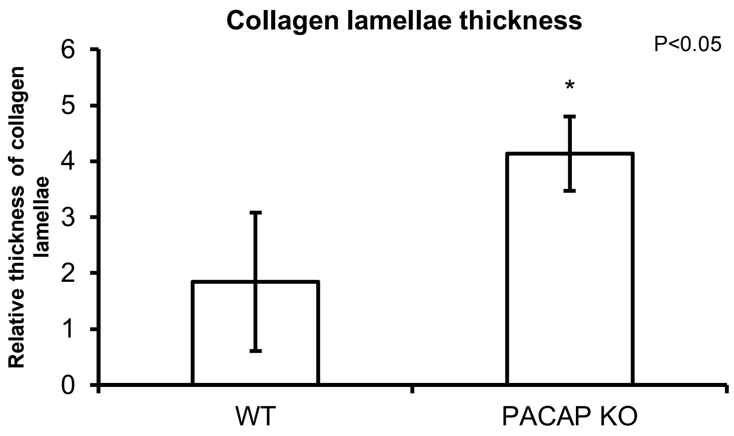

B. Negative controls of collagen type I immunohistochemistry

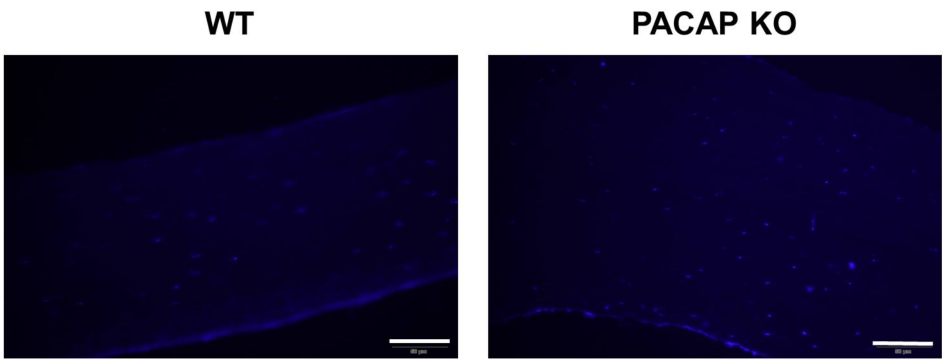

C. Densitometry of RT-PCR reactions

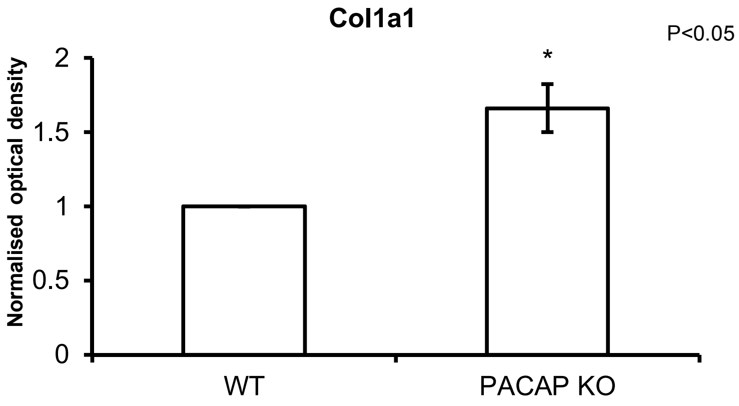

D. Densitometry of Western blot reactions

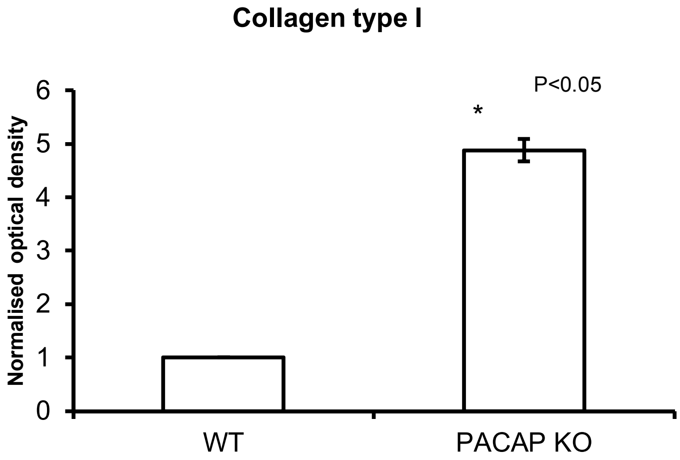

E. Negative control for Col. I. Western blot reactions

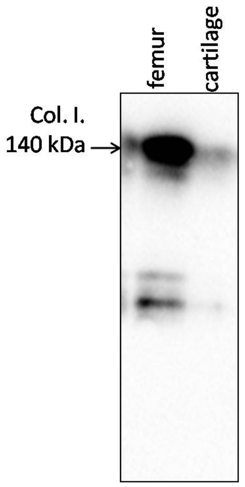

Figure S5. (**A**) Statistical analysis of polarized microscopy of Figure 3 A. Thickness of collagen lamellae was measured and results were normalised to the thickness of WT . (**B**) Negative controls of collagen type I immunohystochemistry. Blue colors represent the nuclei

stained with DAPI, red color shows the aspecific signals of collagen type I. (**C**) and (**D**) Statistical analysis of RT-PCR and Western blot data of Figure 3 C and D. Optical density of limbs was measured by using ImageJ 1.40g freeware. All data are the average of at least three different experiments. Statistical analysis was performed by Student's *t*-test. All data were normalized on Actin and data are expressed as mean ± SEM. Asterisks indicate significant (*P<0.05) alteration of expression as compared to the respective control. (**E**) For negative control of collagen type I Western blot reaction articular cartilage of WT mouse was used. (WT, wild type; PACAP KO).

A. Densitometry of RT-PCR reactions

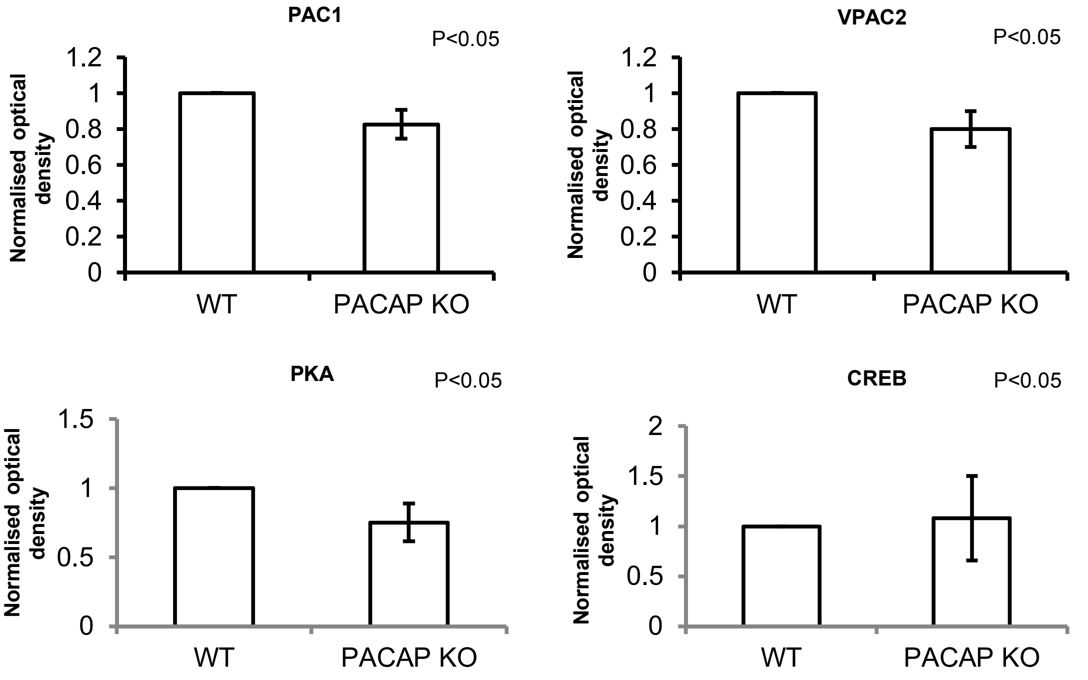

B. Densitometry of Western blot reactions

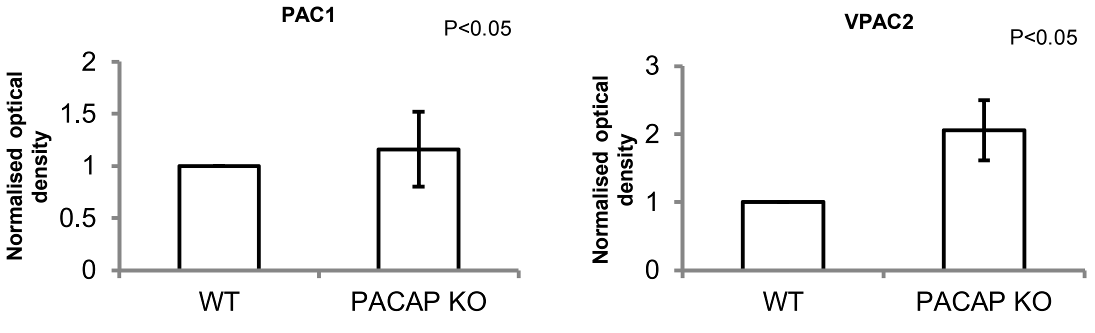

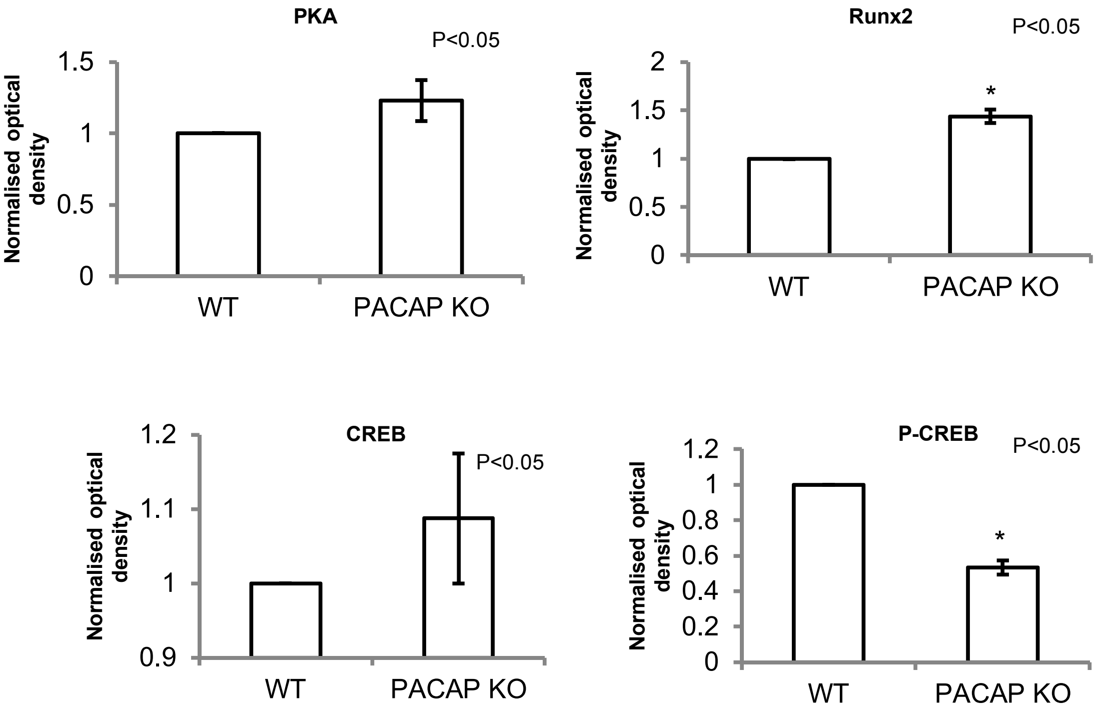

C. Negative controls of Runx2 immunohistochemistry

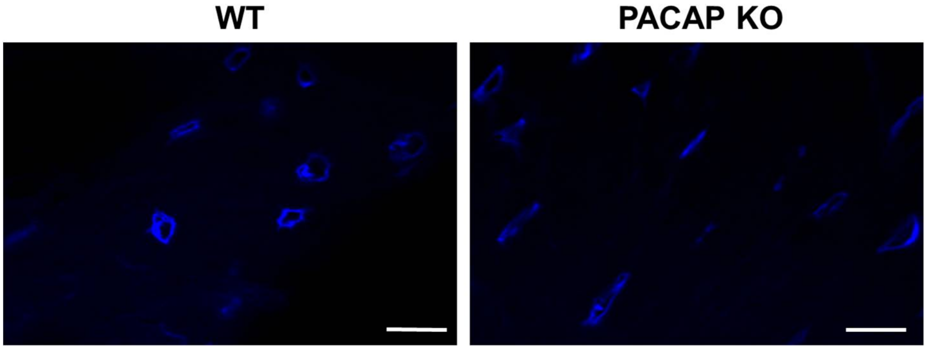

D. Negative or positive controls for PAC1, VPAC1 and VPAC2 Western blot reactions

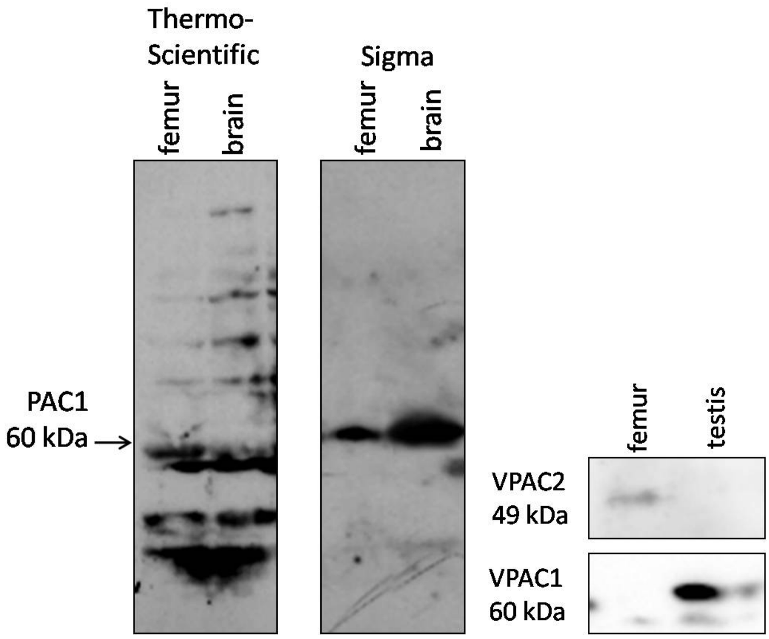

Figure S6. (**A**) and (**B**) Statistical analysis of RT-PCR and Western blot data of Figure 4 A and B. Optical density of limbs was measured by using ImageJ 1.40g freeware. All data are the average of at least three different experiments. Statistical analysis was performed by Student's *t*-test. All data were normalized on Actin and data are expressed as mean ± SEM. Asterisks indicate significant (*P<0.05) alteration of expression as compared to the respective control. (**C**) Negative controls of Runx2 immunohystochemistry. Blue colors represent the nuclei stained with DAPI, red color shows the aspecific signals of Runx2. (**D**) For positive control of PAC1 Western blot reaction brain tissue of WT mouse was used. Antibodies from Thermo-Scientific and Sigma were tested. Testis of WT mice was used to test VPAC1 and VPAC2 antibodies. (WT, wild type; PACAP KO).

A. Densitometry of RT-PCR reactions

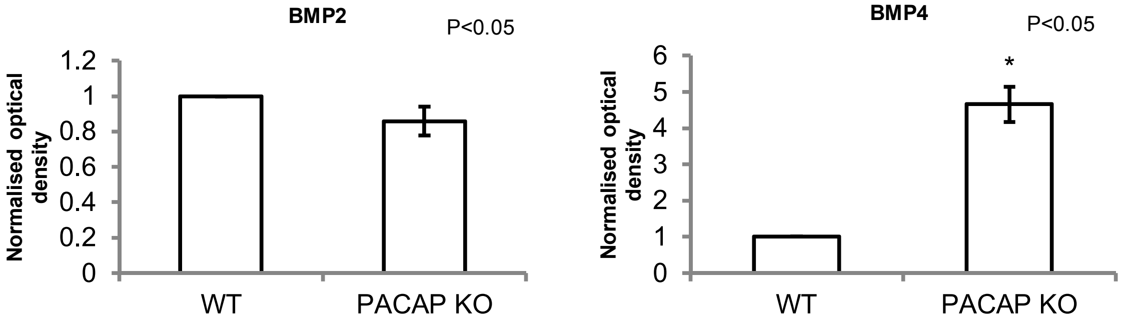

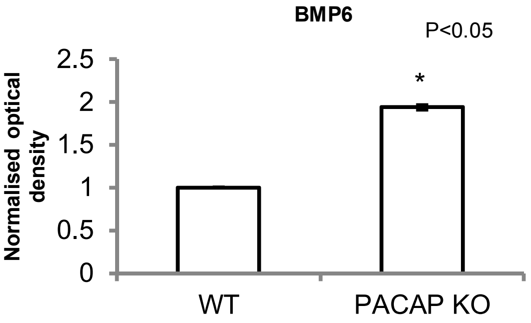

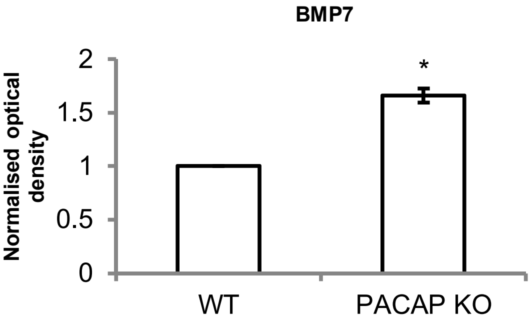

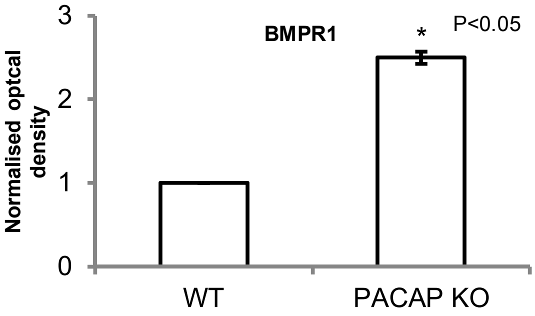

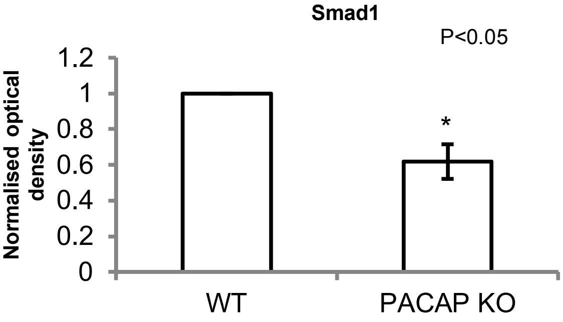

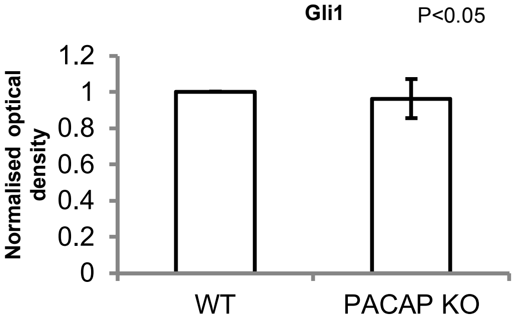

B. Densitometry of Western blot reactions

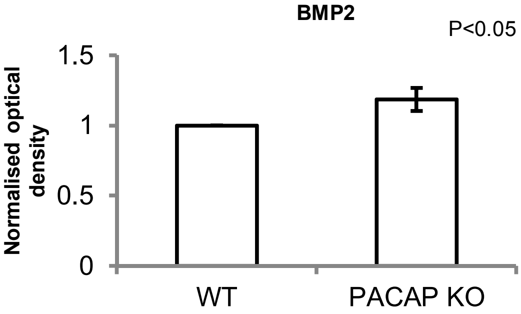

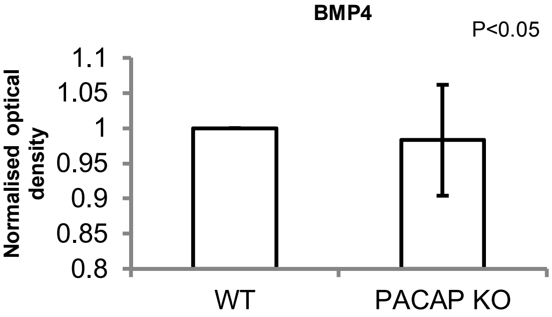

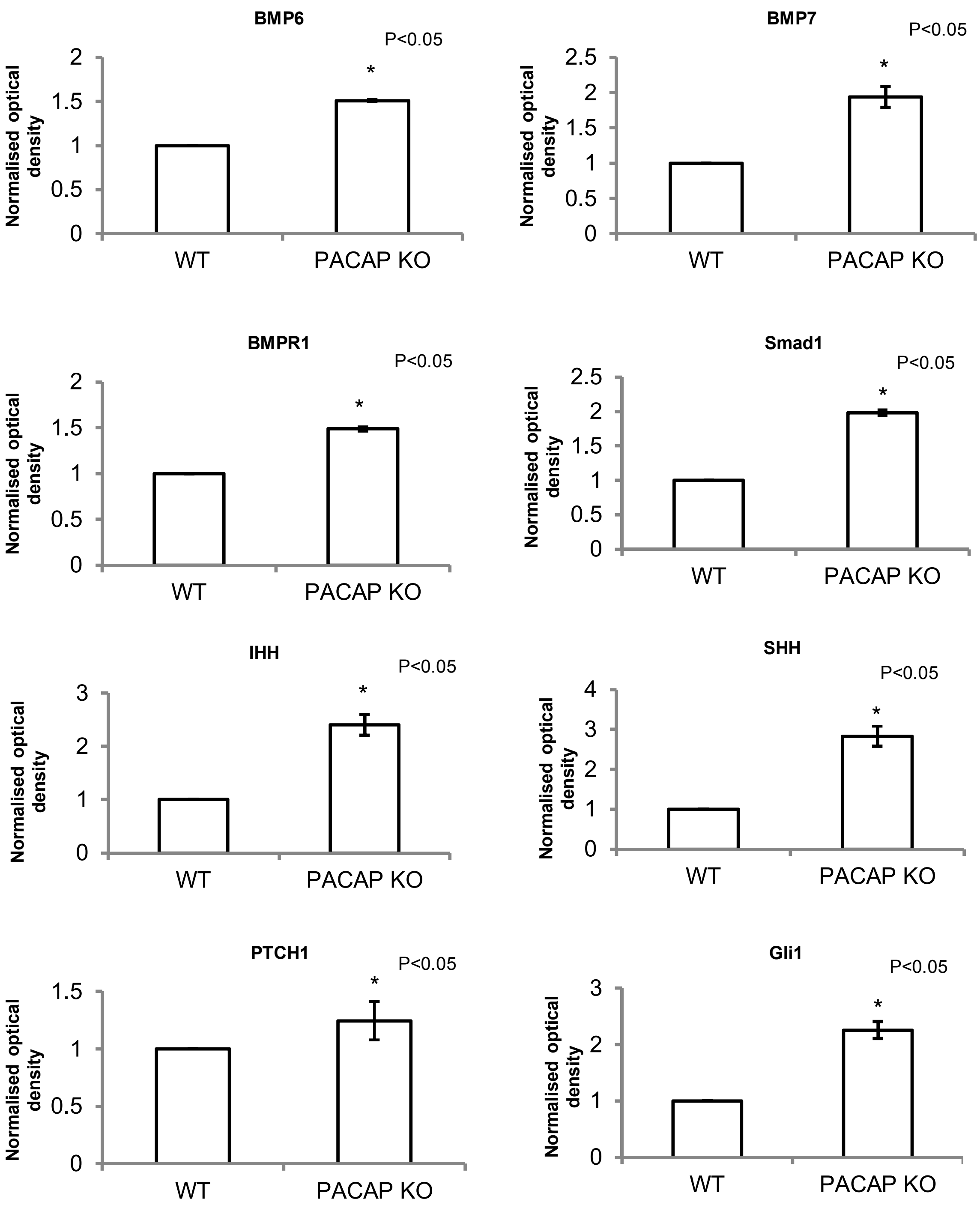

C. Negative controls of Smad1 immunohistochemistry

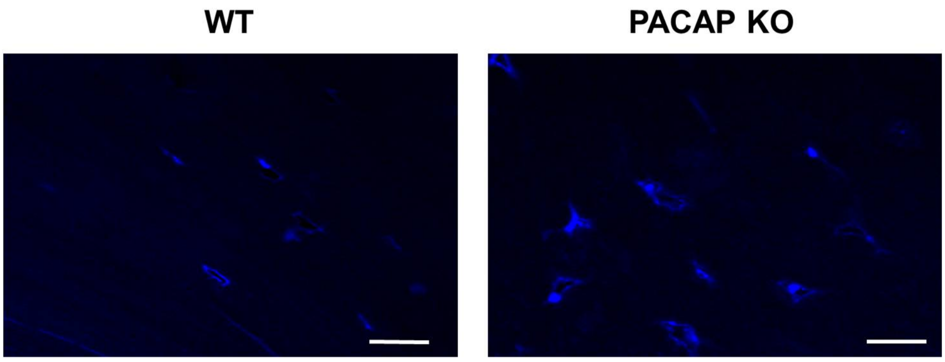

Figure S7. (**A**) and (**B**) Statistical analysis of RT-PCR and Western blot data of Figure 5 A, D and B, E. Optical density of limbs was measured by using ImageJ 1.40g freeware. All data are the average of at least three different experiments. Statistical analysis was performed by Student's *t*-test. All data were normalized on Actin and data are expressed as mean ± SEM. Asterisks indicate significant (*P < 0.05) alteration of expression as compared to the respective control. (**C**) Negative controls of Smad1 immunohystochemistry. Blue colors represent the nuclei stained with DAPI, red color shows the aspecific signals of Smad1. (WT, wild type; PACAP KO).
